# A cohort examination to establish reporting of the remit and function of Trial Steering Committees in randomised controlled trials

**DOI:** 10.1186/s13063-017-2300-1

**Published:** 2017-12-08

**Authors:** Elizabeth J. Conroy, Barbara Arch, Nicola L. Harman, J. Athene Lane, Steff C. Lewis, John Norrie, Matthew R. Sydes, Carrol Gamble

**Affiliations:** 1 MRC North West Hub for Trials Methodology Research, Department of Biostatistics, University of Liverpool, Block F Waterhouse Building, 1-5 Brownlow Street, Liverpool, L69 3GL UK; 2 Clinical Trials Research Centre, University of Liverpool, Liverpool, UK; 3 Bristol Randomised Trials Collaboration, University of Bristol, Bristol, UK; 4 Centre for Population Health Sciences, Edinburgh University, Edinburgh, UK; 5 MRC Clinical Trials Unit at UCL, Institute of Clinical Trials and Methodology, UCL, London, UK; 6 London MRC Hub for Trials Methodology Research, London, UK

**Keywords:** Trials, Oversight committee, Trial Steering Committee, Executive Committee, Clinical Trial, Data Monitoring Committee, Randomised controlled trial

## Abstract

**Background:**

The DAMOCLES project established a widely used Data Monitoring Committee (DMC) Charter for randomised controlled trials (RCTs). Typically, within the UK, the DMC is advisory and recommends to another executive body; the Trial Steering Committee (TSC). Despite the executive role of the TSC, the CONSORT Statement does not explicitly require reporting of TSC activity, although is included as an example of good reporting. A lack of guidance on TSC reporting can impact transparency of trial oversight, ultimately leading to a misunderstanding regarding role and, subsequently, further variation in practice. This review aimed to establish reporting practice of TSC involvement in RCTs, and thus make recommendations for reporting.

**Methods:**

A cohort examination identifying reporting practice was undertaken. The cohort comprised RCTs published in three leading medical journals (the *British Medical Journal*, *The Lancet* and the *New England Journal of Medicine*) within 6 months in 2012 and the full NIHR *HTA Monograph* series. Details of TSC constitution and impact were extracted from main publications and published supplements.

**Results:**

Of 415 publications, 264 were eligible. These were typical in terms of trial design. Variations in reporting between journals and monographs was notable. TSC presence was identified in approximately half of trials (*n* = 144), of which 109 worked alongside a DMC. No publications justified not convening a TSC. When reported, the role of the committee and examples of impact in design, conduct and analysis were summarised.

**Conclusions:**

We present the first review of reporting TSC activity in the published academic literature. An absence of reporting standards with regards to TSC constitution, activity and impact on trial conduct was identified which can influence transparency of reporting trial oversight. Consistent reporting is vital for the benefits and impact of the TSC role to be understood to support adoption of this oversight structure and reduce global variations in practice.

**Electronic supplementary material:**

The online version of this article (doi:10.1186/s13063-017-2300-1) contains supplementary material, which is available to authorized users.

## Background

The DAMOCLES project [1] established a Data Monitoring Committee Charter [2] which has been widely used for randomised controlled trials since 2005. As established within DAMOCLES, the Data Monitoring Committee (DMC) is typically advisory and makes recommendations to another executive body; considered the Trial Steering Committee (TSC). Currently however, no evidence-based Charter exists for TSCs to establish their role and functionality in RCTs.

The MRC Guidelines for Good Clinical Practice (1998) defined a three-committee oversight structure: the day-to-day Trial Management Group, the DMC and the executive TSC [3]. This document provided guidance and a suggested terms of reference for TSCs which has been widely adopted within the UK [4]. However, a need for redevelopment and expansion of these guidelines has been identified [4, 5]. This may have led to these guidelines recently being withdrawn, and subsequently revised, with limited changes mainly concerning responsibilities to funder. The need for the development of expanded universal guidelines remains [3, 6].

Both DAMOCLES [1] and the Consolidated Standards of Reporting Trials (CONSORT) Statement [7], an evidence-based minimum set of recommendations for reporting randomised trials, recommend that trial reports should include information about interim analyses and the data monitoring process. The purpose of CONSORT is to facilitate the complete and transparent reporting of trials and, as such, has been widely adopted by journals to aid critical appraisal and interpretation. However, there is an absence of content focussing on the clinical trial oversight structure and responsibilities for decision-making. While reporting of DMC activity is included within CONSORT as part of reporting interim analyses, and DAMOCLES suggests reporting DMC membership, the reporting of TSC activities is not covered.

The objective of this cohort examination was to establish current practice of reporting of TSC involvement in RCTs, to recommend reporting standards for TSC activity and to identify impact on trial conduct.

## Methods

### Search strategy

EJC searched publications within a 6-month period (1 July 2012 to 31 December 2012) from four sources. Three top general medical journals (the *British Medical Journal* (*BMJ*), *The Lancet* (*Lancet*) and the *New England Journal of Medicine* (*NEJM*)) and within the full UK National Institute for Health Research Health Technology Assessment (NIHR HTA) Monograph series. Journals were selected that are known for endorsing high standards of reporting when publishing RCTs. The NIHR *HTA Monograph* series (*HTA*), a peer-reviewed, open-access journal that publishes full details of a single study funded by the NIHR HTA funding stream. NIHR HTA is a major UK funding body which supports policy-makers such as the National Institute for Health and Care Excellence, the National Screening Committee and the Department of Health [8]. The full NIHR *HTA Monograph* series was searched as opposed to those published within the set timeframe because this series has a suggested word count of 50,000 and so enables more details of the work to be included when compared to a typical peer-reviewed journal and so were considered more likely to provide a comprehensive description of TSC remit and function. Therefore, when summarising examples of reporting, results from journals and monographs are reported separately.

Published RCTs were identified by searching of titles, abstracts and keywords of primary research papers published within the timeframe using the search term random*. When eligibility was unclear a second reviewer (CG) was consulted.

### Inclusion criteria

We included all RCTs publishing main trial results. Articles presenting results of: secondary analyses; preliminary analyses; and additional reports of published RCTs; for example, results of long-term follow-up, were excluded.

### Data extraction and analysis

A Data Extraction Form was designed and piloted by EJC and CG. Data was extracted from all published materials (main trial report and supplementary material when applicable). EJC extracted data on trial design, trial stopping and oversight committee reporting (see Table 1) and entered into an MS Access database. BA independently extracted data from a random 10%, stratified by TSC reporting and source.

**Table 1:** Data Extraction Form

The following details were extracted from eligible articles:
Section 1: Trial details
1.1. Title
1.2. Authors
1.3. Journal
1.4. Funding body
1.5. Year of publication (for HTA series only)
1.6. Rationale of trial
Section 2: Trial design
2.1. Recruitment setting, e.g. primary
2.2. Type of trial, e.g. parallel
2.3. Number of trial arms
2.4. Number of primary outcomes
2.5. Type of primary outcome, e.g. subjective
2.6. Unit of randomisation
2.7. Blinding
(i) Level of blinding
(ii)Reasons provided for non-blinding or level of blinding
Section 3: Sample size
3.1. What was the estimated sample size?
3.2. Was the estimated sample size obtained?
Section 4: Trial stopping
4.1. Did the trial stop early? If yes, give details of why and how this decision was made
Section 5: Oversight committee reporting
5.1. If TSC reported
(i) Are the TSC members listed at the end of the paper?
a. Name of committee
b. Number of members
c. Number of voting members
d. Chair indicated
e. Details regarding the number of members by role and by voting rights if applicable
(ii) Is the TSC discussed in the main body of the paper? If yes, give details
5.2. If DMC reported
(i) Are the DMC members listed at the end of the paper?
a. Name of committee
b. Number of members
c. Number of voting members
d. Chair indicated
e. Details regarding the number of members by role and by voting rights if applicable
(ii) Is the DMC discussed in the main body of the paper? If yes, give details
5.3. If applicable, has the absence of committees been justified? If yes, give details

*DMC* Data Management Committee*, HTA* Health Technology Assessment Monograph series, *TSC* Trial Steering Committee

Quantitative items were analysed using descriptive statistics. Standard statistical software was used throughout (Statistical Analysis Software (SAS 9.1.3; SAS Institute Inc., Cary, NC, USA)). Text extracts from articles were examined using NVivo qualitative data analysis software (QSR International Pty Ltd. Version 10, 2012). EJC and CG identified themes within text items which were used to contextualise and illuminate quantitative results. Extracts are denoted by *(…)* and *(words)* denoted the addition of words or replaced words to aid understanding. Each paper was mapped to a unique project identification number; details of this mapping are given in Additional file 1: Table S1. Due to differences in focus of paper between journal manuscripts and the monograph series, results are presented split by source and, where applicable, split into sections appropriate for the content.

## Results

### Eligible cohort and demographics

**Fig. 1:**
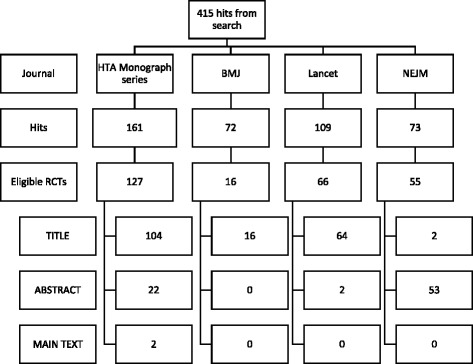
Flowchart of identification of eligible papers

Journals were searched in May 2013. The search returned 415 hits, of which 264 (63.6%) were eligible (127 *HTA*; 16 *BMJ*; 66 *Lancet*; 55 *NEJM*). Figure 1 provides further details. Trial funders were geographically distributed with the majority being of UK (*n* = 161, 61.0%) or USA (*n* = 50, 18.9%) origin. Typically trials were parallel (*n* = 233, 88.3%), two-armed, (*n* = 185, 70.1%) with a pharmaceutical intervention (*n* = 132, 50.0%) and blinded (*n* = 162, 61.4%). Patients were individually randomised (*n* = 237, 90.5%) within the secondary and/or tertiary setting (*n* = 128, 48.5%). Other design features and characteristics, overall and split by source, are summarised in Table 2.

**Table 2:** Trial demographics by journal

			Journal (*N* in cohort)								Total (*N* = 264)	
			*BMJ* (*N* = 16)		*NEJM* (*N* = 55)		*Lancet* (*N* = 66)		*HTA* (*N* = 127)			
			*n*	*n*/*N*%	*n*	*n*/*N*%	*n*	*n*/*N*%	*n*	*n*/*N*%	*n*	*n*/*N*%
Trial rationale	Explanatory		0	0.0	0	0.0	5	7.6	2	1.6	7	2.7
	Pragmatic		6	37.5	2	3.6	10	15.2	88	69.3	106	40.2
	Not specified or clear		10	62.5	53	96.4	51	77.3	37	29.1	151	57.2
Funder origin	Asia		0	0.0	1	1.8	3	4.5	0	0.0	4	1.5
	Japan		0	0.0	1	1.8	2	3.0	0	0.0	3	1.1
	South Korea		0	0.0	0	0.0	1	1.5	0	0.0	1	0.4
	Australia		1	6.3	2	3.6	3	4.5	0	0.0	6	2.3
	Australia		1	6.3	2	3.6	2	3.0	0	0.0	4	1.5
	New Zealand		0	0.0	0	0.0	1	1.5	0	0.0	2	0.8
	Europe		14	87.5	21	38.2	40	60.6	127	100.0	202	76.5
	Belgium		0	0.0	1	1.8	0	0.0	0	0.0	1	0.4
	Denmark		3	18.8	2	3.6	0	0.0	0	0.0	5	1.9
	Finland		1	6.3	0	0.0	0	0.0	0	0.0	1	0.4
	France		0	0.0	2	3.6	5	7.6	0	0.0	7	2.7
	Germany		0	0.0	1	1.8	6	9.1	0	0.0	7	2.7
	Ireland		1	6.3	0	0.0	2	3.0	0	0.0	3	1.1
	Netherlands		2	12.5	1	1.8	2	3.0	0	0.0	5	1.9
	Spain		0	0.0	0	0.0	1	1.5	0	0.0	1	0.4
	Sweden		0	0.0	0	0.0	1	1.5	0	0.0	1	0.4
	Switzerland		1	6.3	4	7.3	3	4.5	0	0.0	8	3.0
	United Kingdom		6	37.5	9	16.4	19	28.8	127	100.0	161	61.0
	Other (European Union)		0	0.0	1	1.8	1	1.5	0	0.0	2	0.8
	North America		1	6.3	30	54.5	20	30.3	0	0.0	51	19.3
	Canada		0	0.0	1	1.8	0	0.0	0	0.0	1	0.4
	United States of America		1	6.3	29	52.7	20	30.3	0	0.0	50	18.9
	Not specified		0	0.0	1	1.8	0	0.0	0	0.0	1	0.4
Recruitment setting	Primary		4	25.0	2	3.6	5	7.6	46	36.2	57	21.6
	Secondary only		2	12.5	2	3.6	5	7.6	17	13.4	26	9.8
	Tertiary only		3	18.8	17	30.9	30	45.5	47	37.0	97	36.7
	Secondary or tertiary (not specified)		0	0.0	0	0.0	2	3.0	3	2.4	5	1.9
	Community		7	43.8	4	7.3	2	3.0	15	11.8	28^a^	10.6
	Emergency		1	6.3	5	9.1	0	0.0	7	5.5	13	4.9
	Hospice		0	0.0	0	0.0	0	0.0	0	0.0	0	0.0
	Social care		0	0.0	0	0.0	0	0.0	0	0.0	0	0.0
	Other setting		0	0.0	1	1.8	1	1.5	0	0.0	2^b^	0.8
	Not clear		0	0.0	32	58.2	22	33.3	9	7.1	63^c^	23.9
Trial design	Parallel		13	81.3	48	87.3	59	89.4	103	81.1	233	88.3
	Sequential		0	0.0	1	1.8	1	1.5	1	0.8	3	1.1
	Crossover		0	0.0	1	1.8	2	3.0	5	3.9	9	3.4
	Cluster		2	12.5	0	0.0	2	3.0	11	8.7	15	5.7
	Factorial		1	6.3	5	9.1	2	3.0	7	5.5	15	5.7
Number of trial arms	2		12	75.0	42	76.4	46	69.7	85	66.9	185	70.1
	3		3	18.8	7	12.7	10	15.2	29	22.8	49	18.6
	4		1	6.3	4	7.3	4	6.1	7	5.5	16	6.1
	5		0	0.0	2	3.6	2	3.0	5	3.9	9	3.4
	6 or more		0	0.0	0	0.0	4	6.1	1	0.8	5^d^	1.9
Type of intervention ^e, f^	Pharmaceutical		3	18.8	41	74.5	54	81.8	34	26.8	132	50.0
	Cellular and gene therapy		0	0.0	1	1.8	2	3.0	0	0.0	3	1.1
	Medical device		0	0.0	5	9.1	5	7.6	16	12.6	26	9.8
	Surgery		0	0.0	3	5.5	1	1.5	10	7.9	14	5.3
	Radiotherapy		0	0.0	1	1.8	3	4.5	9	7.1	13	4.9
	Psychological and behavioural		6	37.5	0	0.0	1	1.5	22	17.3	29	11.0
	Physical		2	12.5	2	3.6	0	0.0	10	7.9	14	5.3
	Complimentary		0	0.0	0	0.0	1	1.5	5	3.9	6	2.3
	Resources and infrastructure		1	6.3	1	1.8	1	1.5	19	15.0	22	8.3
	Other		4	25.0	2	3.6	1	1.5	12	9.4	19	7.2
Number of primary outcomes	1		11	68.8	47	85.5	51	77.3	101	79.5	210	79.5
	2		2	12.5	3	5.5	10	15.2	13	10.2	28	10.6
	3		1	6.3	4	7.3	2	3.0	1	0.8	8	3.0
	4 or more		2	12.5	1	1.8	2	3.0	11	8.7	16	6.1
Primary outcome type	Subjective	1 primary outcome	3	18.8	0	0.0	2	3.0	39	30.7	44	16.7
		2 + primary outcomes	1	6.3	0	0.0	0	0.0	4	3.1	5	1.9
	Objective	1 primary outcome	6	37.5	46	83.6	46	69.7	53	41.7	151	57.2
		2 + primary outcomes	3	18.8	6	10.9	13	19.7	6	4.7	28	10.6
	Both	1 primary outcome	0	0.0	0	0.0	0	0.0	3	2.4	3^g^	1.1
		2 + primary outcomes	1	6.3	2	3.6	1	1.5	14	11.0	18	6.8
	Not clear	1 primary outcome	2	12.5	1	1.8	3	4.5	6	4.7	12	4.5
		2 + primary outcomes	0	0.0	0	0.0	1	1.5	2	1.6	3	1.1
Allocation ratio	Equal e.g. 1:1		13	81.3	46	83.6	55	83.3	115	90.6	229	86.7
	Not equal e.g. 2:1		2	12.5	8	14.5	10	15.2	12	9.4	32	12.1
	Not clear		1	6.3	1	1.8	1	1.5	0	0.0	3	1.1
Unit of randomisation	Individual		12	75.0	52	94.5	62	93.9	113	89.0	239	90.5
	GP practice		0	0.0	0	0.0	1	1.5	9	7.1	10	3.8
	Dyad (e.g. mother-child)		1	6.3	1	1.8	1	1.5	2	1.6	5	1.9
	Other		3	18.8	2	3.6	2	3.0	3	2.4	10^h^	3.8
Blinding	Yes, blinding		11	68.8	37	67.3	45	68.2	69	54.3	162	61.4
	No, not blinded	Justification provided	2	12.5	3	5.5	5	7.6	33	26.0	43^i^	16.3
		Justification not provided	1	6.3	9	16.4	15	22.7	10	7.9	35	13.3
	Not clear		2	12.5	6	10.9	1	1.5	15	11.8	24	9.1

*BMJ* British Medical Journal, *HTA* Health Technology Assessment Monograph series*, NEJM* New England Journal of Medicine

^a^Advertisements in newsletters (*n* = 1); Child and Adolescent Mental Health Services (*n* = 1); Community mental health teams (*n* = 1); Community nurse services (*n* = 1); Community nursing services and community leg ulcers clinics (*n* = 1); Community old age psychiatry services (*n* = 1); Community sources (*n* = 1); Department of Veterans Affairs (*n* = 3); National population registrar (*n* = 1); Community paediatricians (*n* = 1); Registrar (*n* = 3); Schools (*n* = 1); Secondary schools (*n* = 1); University podiatry schools and podiatry clinics (*n* = 1); Vaccination centres in schools (*n* = 1); Villages (*n* = 1); Not specified (*n* = 8)

^b^Other setting: Veterans Affairs Medical Centre (*n* = 1); From other trials (*n* = 1)

^c^Not clear: Adult mental health setting (*n* = 1); Antenatal clinic (*n* = 1); Centres (*n* = 29); Child and Adolescent Mental Health Services (*n* = 1); Child Development Centre (*n* = 1); Clinic site (*n* = 1); Clinical centres (*n* = 1); Clinical sites (*n* = 1), Clinics (*n* = 2); Countries (*n* = 2); European medical centres (*n* = 1); Institutes (*n* = 1); Institutions (*n* = 1); Sites (*n* = 13); Not described (*n* = 7)

^d^Total arms equal to: six (*n* = 2); eight (*n* = 1); nine (*n* = 1); twelve (*n* = 1)

^e^Defined by UK Clinical Research Collaboration Health Research Classification System

^f^Categories not mutually exclusive

^g^Both: Composite (*n* = 3) (Disease improvement calculated from CHAQ, physician’ global assessment of disease activity, parents’ global assessment of overall well-being, number of joints with limited range of movement (ROM), number of active joints and erythrocyte sedimentation rate (*n* = 1)); Foot and Ankle Outcome Score (*n* = 1); Post-operative nausea and vomiting (*n* = 1)

^h^Other units of randomisation: Clinic (*n* = 1); Family (*n* = 2); Hospital (*n* = 1); Household (*n* = 1); Partner (*n* = 1); School (*n* = 1); Village (*n* = 1); Year group (*n* = 1); Paediatric diabetes services (*n* = 1)

^i^Justification for no blinding: Not possible or practical due to nature of intervention or trial design (*n* = 30); Not possible/practical as in practice caused difficulties for patients (*n* = 1); Not possible/practical as shown by other similar trials (*n* = 2); Not possible/practical so cluster randomisation approach used (*n* = 1); Not possible – no additional justification given (*n* = 4); Attempted to blind although were not successful (*n* = 1); Large sample size means that results are not compromise (*n* = 1); Not blinding reflects real practice (*n* = 2); Test for impact of not blinding post trial (*n* = 1)

### Variations in published material

Due to variations in publishing requirements, differences were anticipated between monographs and journals. However, there were clear differences between sources, Key journals were restricted by word count though supplements were often published. While all three journals encouraged protocol and supplementary appendices, this appeared to be endorsed by the *NEJM* only. Further details are provided in Table 3.

**Table 3:** Material reviewed by type and journal

Material	Journal (*N* in cohort)								Total (*N* = 264)	
	*BMJ* (*N* = 16)		*NEJM* (*N* = 55)		*Lancet* (*N* = 66)		*HTA* (*N* = 127)			
	*n*	*n*/*N*%	*n*	*n*/*N*%	*n*	*n*/*N*%	*n*	*n*/*N*%	*n*	*n*/*N*%
Main trial publication	16	100.0	55	100.0	66	100.0	127	100.0	264	100.0
Study protocol as supplementary material	0	0.0	50	90.9	0	0.0	NA	-	50	18.9
Other supplementary material excluding study protocol	5	31.3	52	94.5	50	75.8	NA	-	107	40.5

*BMJ* British Medical Journal, *HTA* Health Technology Assessment Monograph series, *NA* not applicable, *NEJM* New England Journal of Medicine

### Trial oversight committees

Table 4 describes level of TSC and DMC reporting split by publication source.

**Table 4:** Level of Trial Steering Committee (TSC) and Data Management Committee (DMC) reporting split by journal

Committee(s) reported	Journal (*N* in cohort)				Total (*N* = 264)
	*BMJ* (*N* = 16)	*NEJM* (*N* = 55)	*Lancet* (*N* = 66)	*HTA* (*N* = 127)	
TSC and DMC reported	4 25.0%	31 56.4%	19 28.8%	55 43.3%	109 41.3%
*N* papers reporting TSC (*N* papers reporting DMC)					
Acknowledged/listed	2 (2)	19 (23)	14 (15)	52 (50)	87 (90)
Main paper	2 (2)	7 (6)	5 (4)	3 (5)	17 (17)
Supplementary paper	0 (0)	0 (0)	0 (0)	NA	0 (0)
Protocol	0 (0)	5 (2)	0 (0)	NA	5 (2)
TSC only reported	2 12.5%	3 5.5%	1 1.5%	29 22.8%	35 13.3%
*N* papers reporting TSC					
Acknowledged/listed	1	2	1	19	23
Main paper	1	1	0	10	12
Supplementary paper	0	0	0	NA	0
Protocol	0	0	0	NA	0
Reason for no DMC provided	0	0	0	3	3
DMC only reported	0 0.0%	17 30.9%	26 39.4	6 4.7%	49 18.6%
*N* papers reporting DMC					
Acknowledged / listed	0	8	3	5	16
Main paper	0	6	22	1	29
Supplementary paper	0	1	1	NA	2
Protocol	0	2	0	NA	2
Reason for no TSC provided	0	0	0	0	0
Neither committee reported	10 62.5%	4 7.3%	20 30.3%	37 29.1%	71 26.9%
*N* papers					
Reason for no TSC provided	0	0	0	0	0
Reason for no DMC provided	0	2	0	0	2

*BMJ* British Medical Journal, *HTA* Health Technology Assessment Monograph series, *NA* not applicable, *NEJM* New England Journal of Medicine

Publications reporting neither a TSC nor DMC (71/264, 26.9%) varied by source from 7% (*NEJM*, 4/55) to 63% (*BMJ*, 10/16). Only *NEJM* and HTA publications justified no DMC. *NEJM* publications justifying no DMC (*n* = 2) gave reason in the published protocol as a supplement stating that the DMC was not applicable (*NEJM*16) and:

‘A data monitoring committee for efficacy is not required for this study. Data safety monitoring will be conducted on an ongoing basis as detailed in the Safety Review Plan’. (*NEJM*44)

HTA publications justifying not having a DMC (*n* = 3) gave reasons: the trial examined routine therapies (*HTA*67); no interim analyses were planned (*HTA*83) and the trial did not involve a medicinal product (*HTA*109).

Of the 120 trials not explicitly reporting use of a TSC, none justified its absence.

Aside from trials with cellular or gene therapy interventions of which there were few, TSCs were consistently reported regardless of intervention type, from 48% of psychological or behavioural intervention trials (14/29) to 79% physical intervention trials (11/14). DMCs were reported less frequently, from 23% in resources and infrastructure trials (5/22) to 73% in pharmaceutical intervention trials (97/132) or medical device trials (19/26). Trials with neither committee most commonly involved a complimentary intervention (3/6, 50%) or psychological and behavioural (13/29, 45%). Further details are provided in Tables 5 and 6.

**Table 5:** Oversight committee split by intervention type

Intervention type		TSC reported				DMC reported			
		Yes		No		Yes		No	
	*N*	*n*	*n*/*N*%	*n*	*n*/*N*%	*n*	*n*/*N*%	*n*	*n*/*N*%
Pharmaceutical	132	68	51.5%	64	48.5%	97	73.5%	35	26.5%
Cellular and gene therapy	3	0	0.0%	3	100.0%	2	66.7%	1	33.3%
Medical device	26	17	65.4%	9	34.6%	19	73.1%	7	26.9%
Surgery	14	9	64.3%	5	35.7%	9	64.3%	5	35.7%
Radiotherapy	13	7	53.8%	6	46.2%	5	38.5%	8	61.5%
Psychological and behavioural	29	14	48.3%	15	51.7%	11	37.9%	18	62.1%
Physical	14	11	78.6%	3	21.4%	8	57.1%	6	42.9%
Complimentary	6	3	50.0%	3	50.0%	2	33.3%	4	66.7%
Resources and infrastructure	22	13	59.1%	9	40.9%	5	22.7%	17	77.3%
Other	19	13	68.4%	6	31.6%	7	36.8%	12	63.2%

*DMC* Data Management Committee, *TSC* Trial Steering Committee

**Table 6:** Oversight committee split by intervention type

Intervention type		Committee(s) reported							
		TSC and DMC		TSC and no DMC		DMC and no TSC		No TSC and no DMC	
	*N*	*n*	*n*/*N*%	*n*	*n*/*N*%	*n*	*n*/*N*%	*n*	*n*/*N*%
Pharmaceutical	132	60	45.5%	8	6.1%	37	28.0%	27	20.5%
Cellular and gene therapy	3	0	0.0%	0	0.0%	2	66.7%	1	33.3%
Medical device	26	16	61.5%	1	3.8%	3	11.5%	6	23.1%
Surgery	14	6	42.9%	3	21.4%	3	21.4%	2	14.3%
Radiotherapy	13	4	30.8%	3	23.1%	1	7.7%	5	38.5%
Psychological and behavioural	29	9	31.0%	5	17.2%	2	6.9%	13	44.8%
Physical	14	7	50.0%	4	28.6%	1	7.1%	2	14.3%
Complimentary	6	2	33.3%	1	16.7%	0	0.0%	3	50.0%
Resources and infrastructure	22	4	18.2%	9	40.9%	1	4.5%	8	36.4%
Other	19	7	36.8%	6	31.6%	0	0.0%	6	31.6%

*DMC* Data Management Committee, *TSC* Trial Steering Committee

### Trial steering committees

#### Name

Trial Steering Committee was the most common TSC name (61/144, 42%). Other variants were Steering Committee (42/144, 29%); Steering Group (14/144, 10%) and Executive Committee (10/144, 7%). Table 7 shows other variations.

**Table 7:** TSC name split by journal

Name of TSC	Journal (*N* reporting TSC)								Total (*N* = 144)	
	*BMJ* (*N* = 6)		*NEJM* (*N* = 34)		*Lancet* (*N* = 20)		*HTA* (*N* = 84)			
	*n*	*n*/*N*%	*n*	*n*/*N*%	*n*	*n*/*N*%	*n*	*n*/*N*%	*n*	*n*/*N*%
Trial Steering Committee	6	100.0	1	2.9	6	30.0	48	57.1	61	41.7
Steering Committee	0	0.0	18	52.9	12	60.0	12	14.3	42	29.2
Steering Group	0	0.0	0	0.0	0	0.0	14	16.7	14	9.7
Executive Committee	0	0.0	9	26.5	1	5.0	0	0.0	10	6.9
Trial Steering Group	0	0.0	0	0.0	0	0.0	4	4.9	4	2.8
Advisory Committee	0	0.0	2	5.9	0	0.0	0	0.0	2	1.4
Project Steering Group	0	0.0	0	0.0	0	0.0	2	2.4	2	1.4
Study Steering Committee	0	0.0	1	2.9	0	0.0	1	1.2	2	1.4
Clinical Research Organisation	0	0.0	1	2.9	0	0.0	0	0.0	1	0.7
External Protocol Advisory Committee	0	0.0	1	2.9	0	0.0	0	0.0	1	0.7
Monitoring and Steering Committee	0	0.0	1	2.9	0	0.0	0	0.0	1	0.7
Neurology Steering Committee	0	0.0	0	0.0	1	5.0	0	0.0	1	0.7
Scientific Advisory Group	0	0.0	0	0.0	0	0.0	1	1.2	1	0.7
Steering and Advisory Group	0	0.0	0	0.0	0	0.0	1	1.2	1	0.7
Trial Advisory Group	0	0.0	0	0.0	0	0.0	1	1.2	1	0.7

Note: Tabled sorted by total column. *BMJ* British Medical Journal, *HTA* Health Technology Assessment Monograph series, *NEJM* New England Journal of Medicine

#### Membership

Four fifths indicated the number of committee members, varying from half (*BMJ*, 3/6) to 85% (*HTA*, 71/84). The number of members ranged from 2 to 52 with a median of 7. Details are provided in Table 8.

**Table 8:** Membership details provided split by journal

			Journal (*N* reporting TSC)				Total (*N* = 144)
			*BMJ* (*N* = 6)	*NEJM (N* = 34)	*Lancet* (*N* = 20)	*HTA* (*N* = 84)	
Number of members		*n (n/N%)*	3 (50.0)	20 (58.8)	16 (80.0)	71 (84.5)	110 (76.4)
		*mean (SD)*	5 (2)	13 (12)	8 (5)	8 (6)	9 (7)
		*median (IQR)*	5 (3)	9 (9)	6 (5)	6 (5)	7 (6)
		*(min, max)*	(4, 7)	(4, 52)	(3, 22)	(2, 34)	(2, 52)
Chair indicated		*n (n/N%)*	3 (50.0)	20 (58.8)	9 (45.0)	52 (61.9)	84 (58.3)
Expertise of members indicated		*n (n/N %)*	1 (16.7)	10 (29.4)	4 (20.0)	51 (60.7)	66 (45.8)
Expertise	Chief investigator	*n (n/N%)*	0 (0.0)	9 (26.5)	2 (10.0)	13 (15.5)	24 (16.7)
	Trial coordinator	*n (n/N%)*	0 (0.0)	1 (2.9)	0 (0.0)	5 (6.0)	6 (4.2)
	Clinical expert	*n (n/N%)*	0 (0.0)	3 (8.8)	2 (10.0)	41 (48.8)	46 (31.9)
	Statistician	*n (n/N%)*	0 (0.0)	4 (11.8)	2 (10.0)	23 (27.4)	29 (20.1)
	PPI representative	*n (n/N%)*	1 (16.7)	2 (5.9)	0 (0.0)	36 (42.9)	39 (27.1)
	Health economist	*n (n/N%)*	0 (0.0)	0 (0.0)	0 (0.0)	11 (13.1)	11 (7.6)
	Sponsor representative	*n (n/N%)*	0 (0.0)	5 (14.7)	1 (5.0)	2 (2.4)	8 (5.6)
	Industry representative	*n (n/N%)*	0 (0.0)	1 (2.9)	0 (0.0)	1 (1.2)	2 (1.4)

*BMJ* British Medical Journal*, HTA* Health Technology Assessment Monograph series, *IQR* interquartile range, *NEJM* New England Journal of Medicine*, PPI* Patient and Public Involvement*, SD* standard deviation, *TSC* Trial Steering Committee

One hundred and ten publications listed members of which 84 specified a Chair. Other roles or fields of expertise were detailed in almost half of trials with a TSC (66/144). Common representation were clinical (*n* = 46), statistical (*n* = 29) and patient and/or public (PPI) (*n* = 39).

Reporting was unclear with regards to membership and independence.

##### Journal manuscripts

One publication specified that the funder (NIHR) appointed the TSC, stating that members were ‘researchers independent of the study funders, although several have served on their advisory or funding committees’. (*BMJ*12)

One publication indicated voting members when listing members (*Lancet*25).

Ten *NEJM* publications gave details of TSC membership beyond listing members and affiliations. Half of these described sponsor representation, with one specifying this representation as none voting (*NEJM*20).

All 32 (9 *Lancet*, 23 *NEJM*) supplements reporting TSCs listed members and/or affiliations.

Thirteen *NEJM* protocols discussed committee membership. Protocol reporting consisted listing members by role and/or field of expertise (*n* = 10) or by name (*n* = 6), of which three reported both.

##### Monographs

Forty-six gave details of TSC membership beyond listing members and affiliations.

Independence was discussed in 32 publications (32/46, 69.6%), of which 30 specified an independent Chair. Two reported funder input in selecting the Chair.

Members represented the following fields: clinical (*n* = 12), PPI (*n* = 6), statistical (*n* = 3) and health economics (*n* = 1). Including the Chair, the number of independents, discussed in 25 publications, ranged from two to six (most commonly three, *n* = 17). Non-independent members, discussed in three publications, specified statistical (*n* = 3) and health economics (*n* = 2) representation.

Thirty-one listed members without specifying independence. Represented fields were Patient and Public Involvement (PPI) (*n* = 21), clinical (*n* = 12), statistical (*n* = 9), health economics (*n* = 3) and funder (*n* = 2). Three allowed observers at meetings.

#### Meetings

##### Journal manuscripts

One publication discussed meeting frequency (bimonthly meetings (*BMJ*15)). One stated yearly reports were circulated (*Lancet*3) possibly indicating yearly meetings.

Seven supplementary protocols provided TSC meeting information. Four gave the frequency of these, ranging from monthly to annually. Two were unclear stating meetings were held periodically. Others reported that meetings held by teleconference (*n* = 2) and gave insight to report contents (*n* = 1).

##### Monographs

Twenty-five publications (25/144) discussed meeting frequency or timing. Timings, when specified, were biyearly (*n* = 12); yearly (*n* = 7); quarterly (*n* = 2) and as required (*n* = 1). Three gave meeting dates and another three gave the total number of meetings. One specified the length of TSC meetings as 80–100 min. Four indicated timing of first meeting as prior to (*n* = 2) or at (*n* = 1) trial commencement and before recruitment (*n* = 1)

#### Role

##### Journal manuscripts

Eleven *Lancet* and 17 *NEJM* publications indicated role or responsibility. No *BMJ* articles discussed role. One supplementary appendix reporting TSCs (1/23 *NEJM*) gave insight into TSC role.

**Design and oversight**

Four *Lancet* publications reported involvement in trial design, specifically the committee designing the study with sponsor (2/4), under surveillance of the DMC (1/4), or supervising the design (1/4). Eleven *NEJM* publications reported involvement in design, wherein the role encompassed overseeing (2/11), being responsible for (3/11) and/or involvement in (7/11) trial design, with one specifying that this was independent of the sponsor.

Three *Lancet* publications reported the TSC oversee the trial, of which one stated that the TSC supervised operations. Of the nine *NEJM* publications discussing oversight and conduct, five stated that the TSC oversees the trial (5/9), of which two required that they oversee conduct specifically (2/9). A further four stated that the TSC was responsible for study conduct (4/9).

One supplementary appendix reporting TSCs (1/32, 1/9 *Lancet*) indicated operational oversight role, specifically:

‘The Steering Committee was responsible for overseeing the scientific and operational aspect of the study’. (*Lancet*49)

Thirteen protocols discussed a role in oversight and trial design: the role encompassed monitoring (*n* = 5) and oversight of various aspects of the study (*n* = 5). The TSC had a responsibility or participated in trial conduct (*n* = 8) and design (*n* = 5). One stated that the TSC was independent.

**Decision-making**

Two *Lancet* publications reported a decision-making role, stating that the TSC could decide on study continuation. While no *NEJM* main papers reported the decision-making role, nine protocols did. Five explicitly defined the TSC as the decision-making body, the remaining four opted to provide examples of TSC decisions, such as altering sample size (*n* = 2), patient withdrawals (*n* = 1) and trial stopping (*n* = 1).

**Contribution to trial documentation, data and analysis**

Seven *Lancet* publications involvement in trial documentation, stating that the TSC wrote or contributed to the final report (3/7), made the decision to publish (2/7), or both (1/7). The TSC coordinated and resolved doubts in interpretation in the protocol in another. Analysis and data involvement was reported in five studies, three of these stated the TSC had full access to data (3/5), one supervised the analysis (1/5) and three interpreted the results (one stating that this was done independently). Another stated:

‘The (*TSC*) vouched for the completion and accuracy of the data gathering and analysis’. (*Lancet*6)

Seven *NEJM* publications reported TSC involvement in trial documentation, in contributing to writing the manuscript (4/7) and developing the protocol (3/7), of which one developed this with the sponsor. Four reported TSC involvement in trial data, specifically the TSC had full access to data (1/4) and was involved with data collection (1/4); interpretation (1/4) and analysis (1/4). In seven, the committee made the decision to publish, one stated that the TSC vouches for integrity and completeness of the data and six stated:

‘The (*TSC*) vouches for the accuracy and completeness of the data and the analysis and the fidelity of the study to the protocol’.

One supplementary appendix reporting TSCs (1/32, 1/23 NEJM) gave insight into role. Fifteen protocols discussed TSC input into trial documentation, data and analysis. The TSC was reported to have an input in publications (*n* = 13), the protocol (*n* = 7) and side studies (*n* = 4).

**Communication**

No publications specified communication between TSC and other committees.

One appendix reporting TSCs (1/32) discussed communication, stating:

‘(*the DMC*) made recommendations to the Steering Committee regarding endpoint analysis or potential safety concerns’. (*Lancet*49)

Eight protocols discussed the TSC communicating with the DMC (*n* = 7) and a Critical Event Committee (*n* = 1).

##### Monograph

Forty monographs described role.

**Design and oversight**

Twenty-seven monographs described an oversight role, generally (11/27) or, more specifically, overseeing progress towards interim and overall objectives (8/27) or study progress as a whole (8/27). Three stated independent oversight and two that his was on behalf of the sponsor.

Generic definitions of TSC monitoring were provided in nine monographs. This was done in accordance with the MRC guidelines (*n* = 6), Good Clinical Practice (*n* = 1) and in two:

‘The (*TSC*) ensured that the rights, safety and well-being of the trial participants were the most important considerations and prevailed over the interests of science and society’.

**Decision-making**

Role in decision-making was reported in seven monographs. Examples were about how the study is run (*n* = 2); premature closing (*n* = 5); and how pilot data will inform the main trial (*n* = 2).

**Contribution to trial documentation, data and analysis**

Twelve had the TSC review trial specific documentation. Specifically, the statistical analysis plan (*n* = 8) and protocol (*n* = 5).

Ten stated that the TSC review external information and five had authority over the publication strategy. One had the TSC approve further analysis and one approved additional studies.

**Communication**

The committee received recommendations from the DMC in six monographs, informed funders on trial progress in three, advised funders in one and liaised between the DMC and the Trial Management Group (TMG) in another. One stated that TSC responsibility in resolving disputes between PIs and another had the DMC as a subgroup of the TSC.

#### Activities

##### Journal manuscripts

TSC activity having impact on trial design, conduct and analysis was reported in 12 publications (3 *BMJ*; 7 *Lancet*; 2 *NEJM*) with a total 14 examples reported (4 *BMJ*; 8 *Lancet*; 2 *NEJM*). No activities were reported in supplementary protocols.

**Design**

Twelve (12/14; 3/4 *BMJ*; 7/8 *Lancet*; 2/2 *NEJM*) publications reported TSC activity impacting trial design. Reported activities varied within journal.

The *BMJ* reported TSC involvement changing the sample size (*n* = 2) and primary outcome (*n* = 1):

‘Before the start of recruitment and data collection, we changed the primary outcome to the reported quit attempt measure, which is predictive of eventual cessation. This followed expert advice from the Trial Steering Committee on the basis of smoking cessation research and approval from the Data Monitoring Committee’. (*BMJ*15)

All seven Lancet decisions regarded early stopping. In five, the TSC decided to stop recruitment, of which three reported that this decision was based on DMC recommendations. Others reported the TSC deciding to close recruitment to a trial arm (*n* = 1) and make the decision to continue recruitment following an interim analysis (*n* = 1):

‘Without revealing any results, the DSMB recommended to the Executive Committee and sponsor that the trial continue to the original pre-planned sample size. The basis for this recommendation was that, because of the rapid enrolment at the time of the interim analysis, there was insufficient 90-day data to assess the secondary endpoints, although there were no safety concerns. The Executive Committee and sponsor accepted the DSMB recommendation to continue enrolment, but remained masked to all study results’. (*Lancet*36)

Within the *NEJM* publications reporting TSC activity relating to trial design (*n* = 2), the committee established when patients could have their dose tapered (*NEJM*42) and when crossover could be permitted:

‘The independent Data and Safety Monitoring Committee and Study Steering Committee concluded that both progression-free survival and overall survival were significantly longer in the trametinib group than in the chemotherapy group and that immediate crossover to trametinib should be permitted’. (*NEJM*23)

One supplementary appendix reported TSCs (1/32) publishing the letter of recommendation from the DMC to the TSC requesting one arm be closed due to accruing safety data (*NEJM*38), this was consistent with the main publication wherein the trial was prematurely stopped.

**Conduct**

No publication reporting TSC activity related to trial conduct.

**Analysis**

Two (2/14; 1/4 *BMJ*; 1/8 *Lancet*) publications reported TSC activity impacting analysis.

One described the TSC deciding to write a new SAP (*BMJ*3) Another reported the TSC determining exclusions from analysis and imputations for deaths or drop outs (*Lancet*10).

##### Monographs

Thirty-seven monographs reported 60 examples of TSC activity.

**Design**

Thirty-four examples of TSC activity impacting trial design was reported in 23 monographs.

Activities included the TSC changing the entry criteria (*n* = 7); endpoints or outcomes (*n* = 7); sample size (*n* = 2); and randomisation ratio (*n* = 1). Another reported the TSC closing a treatment arm (*n* = 1). Others reported TSC impact in defining the study design (*n* = 6) and recruitment period (*n* = 2).

Most significantly, the TSC made the decision to close the trial in five studies, as in *HTA*238:

‘In a meeting of the Trial Steering Committee, it was accepted that it would not be viable to proceed with the trial and the formal procedure for closure (including notification of MHRA and MREC) was initiated in May 2005’. (*HTA*75)

One monograph discussed the TSC overriding the recommendations of the DMC to close the trial:

‘Although the DMC recommended continuation of recruitment into FOOD following their meeting in 2002, the Steering Committee took the decision to stop recruitment on 31 July 2003’. (*HTA*88)

**Conduct**

Fifteen examples of TSC activity impacting trial conduct was given in 14 monographs.

Examples of conduct were where the TSC: had input in determining the data collection process (4/14); considered consent issues (1/14) and input into safety issues; for example, such as reviewing death data (1/14) and determining serious adverse event (SAE) data requirements (3/14).

In three (3/14) the TSCs made the decision to changing treatment regimens; for example:

‘The project Steering Group determined that (*a prescription*) was inappropriate to a pragmatic study of this kind. It was agreed that the outcome would be more likely to represent the likely outcome of introducing TUVP if staff were to manage patients according to existing norms’. (*HTA*30)

Others (3/14) changed the recruitment procedure, for example:

‘On reflection and discussion of these issues, the research team and the Trial Steering Committee members felt that some of these issues could have been addressed (…). They concluded that many of the problems encountered were a direct consequence of the changes in research governance and ethical procedures that prevent members of the research team approaching patients directly, but instead place the burden of recruiting patients on busy primary care professionals’. (*HTA*75)

Analysis

Ten examples of TSC activity impacting analysis was given in ten monographs.

The TSC impacted the analysis plan in seven monographs (7/10). Examples were: determining variables for regression model (2/7), defining equivalence limits (1/7), removing previously planned subgroup analyses (1/7), deciding on the analysis approach to be used, e.g. intention-to-treat (2/7) or suggesting additional analysis:

‘Finally, at the request of the TSC, a further exploratory analysis to examine the interactive effect of age on the effectiveness of MRI compared with no MRI was conducted’. (*HTA*111)

Other activities were the TSC determining protocol violations (1/10) and making the decision to unblind the trial team (2/10), for example:

‘The identification of treatments was established by code break in the presence of the Chief Investigator, Trial Statistician and Trial Coordinator on 20 March 2007 by agreement with the TSC and DMEC’. (*HTA*104)

## Discussion

The extent of the adoption of a TSC, the committee with a majority of independent members to whom the DMC make their recommendations, for trial oversight outside of the UK is unknown. Within the UK the establishment of a TSC is required by a number of major public funders yet, despite this, there is an absence of reporting standards regarding their constitution, activities and impact on trial conduct.

This paper aimed to provide the first review of reporting of TSC activity by reviewing published academic literature from within and outside the UK. Determining the role and contribution of this executive oversight committee was limited by a lack of reporting and, in particular, clear indication of whether this committee included a majority of, or even any, independent members. It was often unclear whether the TSC being referenced was in fact the TSC or the TMG, the committee with heavy intellectual and practical investment in governing the day-to-day running of the trial. In trials where no major decisions need to be made, this may seem unimportant. In trials where DMC recommendations are not actioned then it is of increasing importance to understand the extent of the vested interests of the committee considering those recommendations. In the cohort reported here one such example was noted where the DMC recommended trial continuation but the TSC decided to close the trial. Arguably, this may be more concerning if this was the other way around; however, poor reporting standards will obscure this occurrence (*HTA*88).

When interpreting these results, it is important to consider the limitations which include a restriction of the cohort to the top medical journals and the NIHR *HTA Monograph* series. While this has the advantage publishing international trials, it may also be argued that these are of higher quality. The poor standards observed within this cohort, therefore, may be lower elsewhere. The timeframe of this cohort also prevented consideration of changes over time; however, given the absence of attention received to this important role and its reporting standards this is unlikely. As previously discussed, the extent of the adoption of this oversight committee structure is not known for trials outside of UK and little is known about industry-funded and sponsored trials.

For TSCs to be accepted as good practice globally, it is important that the benefits and impact of such a committee are reported. This paper has highlighted the need to improve reporting of TSCs and, in particular, clarify the independence, or otherwise, of its members. Despite the academic literature search being conducted in 2012 there has been no advancement in reporting guidelines in this area and the situation remains unchanged. Current reporting recommendations for DMCs [1, 2] could be used as a starting point with focus on decisions made by the TSC.

One challenge of writing a report of a clinical trial is including pertinent information within word limits set by journals. It is often a balance of what can be left out without jeopardising quality. However, clarity of reporting on decision-making processes would seem essential given the potential for bias. With the availability of supplementary material, researchers must make this information publicly accessible. This would greatly aid the transparency of clinical trials and allow understanding of stakeholder involvement in decisions made.

## Conclusions

This cohort examination provides the first examination of reporting practice of Trial Steering Committee involvement in randomised controlled trials. A lack of reporting standards has been identified, resultantly understanding the benefits and impact of the TSC role using the academic literature is challenging. Developing reporting guidelines is essential to aid determining the role and contribution of this executive oversight committee. This would improve reporting standards, which would greatly aid the transparency of clinical trials and allow understanding of stakeholder involvement in decision-making.

## Additional file

Additional file 1: Table S1. Project identification numbers of included articles. Table listing all trials in cohort. (DOCX 48 kb)

## Abbreviations

*BMJ*: *British Medical Journal*
DMC: Data Monitoring Committee
*NEJM*: *New England Journal of Medicine*
NICE: National Institute for Health and Care Excellence
NIHR HTA: National Institute for Health Research Health Technology Assessment
TMG: Trial Management Group
TSC: Trial Steering Committee
UK: United Kingdom

## References

[1] Grant A, Altman D, Babiker A (2005). Issues in data monitoring and interim analysis of trials. Health Technol Assess.

[2] DAMOCLES Study Group (2005). A proposed charter for clinical trial data monitoring committees: helping them to do their job well. Lancet..

[3] MRC Guidelines for Good Clinical Practice in Clinical Trials. Online referencing*.* 1998. Available at: https://www.mrc.ac.uk/. Accessed 15 Sept 2017.

[4] Conroy EJ, Harman NL, Lane JA (2015). Trial Steering Committees in randomised controlled trials: a survey of registered clinical trials unites to establish current practice and experiences. Clin Trials.

[5] Harman NL, Conroy EJ, Lewis SC (2015). Exploring the role and function of Trial Steering Committees: results of an expert panel meeting. Trials..

[6] MRC Guidelines for Management of Global Health Trials. Online referencing. 2017. Available at https://www.mrc.ac.uk/. Accessed 1 Oct 2017.

[7] Schulz KF, Altman DG, Mohor D (2010). CONSORT 2010 Statement: updated guidelines for reporting parallel group randomised trials. BMJ..

[8] Royle P, Waugh N (2013). Bibliometrics of NIHR HTA monographs and their related journal articles. BMJ Open..

