# Supplementary material for: A cohort examination to establish reporting of the remit and function of Trial Steering Committees in randomised controlled trials
Source: Trials. 2017 Dec 8;18:590. doi: 10.1186/s13063-017-2300-1 (PMC5723080; doi:10.1186/s13063-017-2300-1)
Supplement: Additional file 1: Table S1. — Project identification numbers of included articles. Table listing all trials in cohort. (DOCX 48 kb) [file 13063_2017_2300_MOESM1_ESM.docx]

**Additional file 1: Table S1: Project identification numbers of included articles**

| **ID** | **Reference** |
| --- | --- |
| BMJ1 | Bischoff EWMA, Akkermans R, Bourbeau J, van Weel C, Vercoulen JH, et al. Comprehensive self management and routine monitoring in chronic obstructive pulmonary disease patients in general practice: randomised controlled trial. BMJ 2012;345:e7642. |
| BMJ2 | Bowen A, Hesketh A, Patchick E, Young A, Davies L, et al. Effectiveness of enhanced communication therapy in the first four months after stroke for aphasia and dysarthria: a randomised controlled trial. BMJ 2012;345:e4407. |
| BMJ3 | Chappell LC, Gurung V, Seed PT, Chambers J, Williamson C, et al. Ursodeoxycholic acid versus placebo, and early term delivery versus expectant management, in women with intrahepatic cholestasis of pregnancy: semifactorial randomised clinical trial. BMJ 2012;344:e3799. |
| BMJ4 | Clemson L, Fiatarone Singh MA, Bundy A, Cumming RG, Manollaras K, et al. Integration of balance and strength training into daily life activity to reduce rate of falls in older people (the LiFE study): randomised parallel trial. BMJ 2012;345:e4547. |
| BMJ5 | Hollands GJ, Whitwell SCC, Parker RA, Prescott NJ, Forbes A, et al. Effect of communicating DNA based risk assessments for Crohn’s disease on smoking cessation: randomised controlled trial. BMJ 2012;345:e4708. |
| BMJ6 | Ibler KS, Jemec GBE, Diepgen TL, Gluud C, Hansen JL, et al. Skin care education and individual counselling versus treatment as usual in healthcare workers with hand eczema: randomised clinical trial. BMJ 2012;345:e7822. |
| BMJ7 | Leinonen MK, Nieminen P, Lönnberg S, Malila N, Hakama M, et al. Detection rates of precancerous and cancerous cervical lesions within one screening round of primary human papillomavirus DNA testing: prospective randomised trial in Finland. BMJ 2012;345:e7789 |
| BMJ8 | Miller G, Luo R, Zhang L, Sylvia S, Shi Y, Foo P, et al. Effectiveness of provider incentives for anaemia reduction in rural China: a cluster randomised trial. BMJ 2012;345:e4809. |
| BMJ9 | Morthorst B, Krogh J, Eriangsen A, Alberdi F, Nordentoft M. Effect of assertive outreach after suicide attempt in the AID (assertive intervention for deliberate self harm) trial: randomised controlled trial. BMJ 2012;345:e4972. |
| BMJ10 | Schierbeck LL, Rejnmark L, Tofteng CL, Stilgre L, Eiken P, et al. Effect of hormone replacement therapy on cardiovascular events in recently postmenopausal women: randomised trial. BMJ. 2012 Oct 9;345:e6409. doi: 10.1136/bmj.e6409. |
| BMJ11 | Stallard P, Sayal K, Phillips R, Taylor JA, Spears M, et al. Classroom based cognitive behvioural therapy in reducing symptoms of depression in high risk adolescents: pragmatic cluster randomised controlled trial. BMJ 2012;345:e6058. |
| BMJ12 | Stewart S, Carrington MJ, Swemmer CH, Anderson C, Kurstjens NP, et al. Effect of intensive structured care on individual blood pressure targets in primary care: multicentre randomised controlled trial. BMJ 2012;345:e7156. |
| BMJ13 | van den Broek IV, van Bergen JE, Brouwers EE, Fennema JS, Göts HM, et al. Effectiveness of yearly, register based screening for chlamydia in the Netherlands: controlled trial with randomised stepped wedge implementation. BMJ. 2012 Jul 5;345:e4316. doi: 10.1136/bmj.e4316. |
| BMJ14 | Waldoff FB, Buss DV, Eckermann A, Rasmussen MLH, Keilding N, et al. Efficacy of psychosocial intervention in patients with mild Alzheimer’s disease: the multicentre, rater blinded, randomised Danish Alzheimer Intervention Study (DAISY). BMJ 2012;345:e4693. |
| BMJ15 | Walsh JM, McGowan CA, Mahony R, Foley ME, McAuliffe FM. Low glycaemic index diet in pregnancy to prevent macrosomia (ROLO study): randomised controlled trial. BMJ 2012;345:e5605. |
| BMJ16 | Walter FM, Morris HC, Humphys E, Hall PN, Prevost AT, et al. Effect of adding a diagnostic aid to best practice to manage suspicious pigmented lesions in primary care: randomised controlled trial. BMJ 2012;345:e4110. |
| HTA1 | Allsup S, Gosney M, Haycox A, Regan M. Cost–benefit evaluation of routine influenza immunisation in people 65–74 years of age. Health Technol Assess. 2003;7(24):iii-x, 1-65. |
| HTA2 | Appleton RE, Jones AP, Gamble C, Williamson PR, Wiggs L, et al. The use of MElatonin in children with Neurodevelopmental Disorders and impaired Sleep: a randomised, double-blind, placebo-controlled, parallel study (MENDS). Health Technol Assess. 2012;16(40):i-239. doi: 10.3310/hta16400. |
| HTA3 | Banerjee S, Hellier J, Romeo R, Dewey M, Knapp M, et al. Study of the use of antidepressants for depression in dementia: the HTA -SADD trial – a multicentre, randomised, double-blind, placebo-controlled trial of the clinical effectiveness and cost-effectiveness of sertraline and mirtazapine. Health Technol Assess. 2013 Feb;17(7):1-166. doi: 10.3310/hta17070. |
| HTA4 | Baxter-Jones ADG, Helms PJ, Russell G, Grant A. Early asthma prophylaxis, natural history, skeletal development and economy (EASE): a pilot randomised controlled trial. Health Technol Assess. 2000;4(28):1-89. |
| HTA5 | Boland A, Haycox A, Bagust A, Fitzsimmons L. A randomised controlled trial to evaluate the clinical and costeffectiveness of Hickman line insertions in adult cancer patients by nurses. Health Technol Assess. 2003;7(36):iii, ix-x, 1-99. |
| HTA6 | Bowen A, Hesketh A, Patchick E, Young A, Davies L, et al. Clinical effectiveness, cost effectiveness and service users’ perceptions of early, well-resourced communication therapy following a stroke: a randomised controlled trial (the ACT NoW Study). Health Technol Assess. 2012 May;16(26):1-160. doi: 10.3310/hta16260. |
| HTA7 | Bowns IR, Collins K, Walters SJ, McDonagh AJG. Telemedicine in dermatology: a randomised controlled trial. Health Technol Assess. 2006 Nov;10(43):iii-iv, ix-xi, 1-39. |
| HTA8 | Boyle J, McCartney E, Forbes J, O’Hare A. A randomised controlled trial and economic evaluation of direct versus indirect and individual versus group modes of speech and language therapy for children with primary language impairment. Health Technol Assess. 2007 Jul;11(25):iii-iv, xi-xii, 1-139. |
| HTA9 | Bradbury AW, Adam DJ, Bell J, Forbes JF, Fowkes FGR, et al. Multicentre randomised controlled trial of the clinical and cost-effectiveness of a bypass-surgery-first versus a balloonangioplasty- first revascularisation strategy for severe limb ischaemia due to infrainguinal disease. The Bypass versus Angioplasty in Severe Ischaemia of the Leg (BASIL) trial. Health Technol Assess. 2010 Mar;14(14):1-210, iii-iv. doi: 10.3310/hta14140. |
| HTA10 | Brown LC, Powell JT, Thompson SG, Epstein DM, Sculpher MJ, et al. The UK EndoVascular Aneurysm Repair (EVAR) trials: randomised trials of EVAR versus standard therapy. Health Technol Assess. 2012;16(9):1-218. doi: 10.3310/hta16090 |
| HTA11 | Bryan S, Weatherburn G, Bungay H, Hatrick C. The cost-effectiveness of magnetic resonance imaging for investigation of the knee joint. Health Technol Assess. 2001;5(27):1-95. |
| HTA12 | Caine N, Sharples LD, Hollingworth W. A randomised controlled crossover trial of nurse practitioner versus doctor-led outpatient care in a bronchiectasis clinic. Health Technol Assess. 2002;6(27):1-71. |
| HTA13 | Campbell MK, Skea ZC, Sutherland AG, Cuthbertson BH, Entwistle VA, et al. Effectiveness and cost-effectiveness of arthroscopic lavage in the treatment of osteoarthritis of the knee: a mixed methods study of the feasibility of conducting a surgical placebo-controlled trial (the KORAL study). Health Technol Assess. 2010 Jan;14(5):1-180. doi: 10.3310/hta14040. |
| HTA14 | Chalder M, Wiles NJ, Campbell J, Hollinghurst SP, Searle A, et al. A pragmatic randomised controlled trial to evaluate the cost-effectiveness of a physical activity intervention as a treatment for depression: the treating depression with physical activity (TREAD) trial. Health Technol Assess. 2012;16(10):1-164, iii-iv. doi: 10.3310/hta16100. |
| HTA15 | Charlesworth G, Shepstone L, Wilson E, Thalanany M, Mugford M, et al. Does befriending by trained lay workers improve psychological well-being and quality of life for carers of people with dementia, and at what cost? A randomised controlled trial. Health Technol Assess. 2008 Mar;12(4):iii, v-ix, 1-78. |
| HTA16 | Cochrane T, Davey RC, Matthes Edwards SM. Randomised controlled trial of the cost-effectiveness of water-based therapy for lower limb osteoarthritis. Health Technol Assess. 2005 Aug;9(31):iii-iv, ix-xi, 1-114. |
| HTA17 | Cockayne S, CurranM , Denby G, Hashmi F, Hewitt C, et al. EVerT: cryotherapy versus salicylic acid for the treatment of verrucae – a randomised controlled trial. Health Technol Assess. 2011 Sep;15(32):1-170. doi: 10.3310/hta15320. |
| HTA18 | Cooke MW, Marsh JL, Clark M, Nakash R, Jarvis RM, et al. Treatment of severe ankle sprain: a pragmatic randomised controlled trial comparing the clinical effectiveness and cost-effectiveness of three types of mechanical ankle support with tubular bandage. The CAST trial. Health Technol Assess. 2009 Feb;13(13):iii, ix-x, 1-121. doi: 10.3310/hta13130. |
| HTA19 | Crawford MJ, Killaspy H, Barnes TR, Barrett B, Byford S, et al. Group art therapy as an adjunctive treatment for people with schizophrenia: a randomised controlled trial (MATISSE). Health Technol Assess. 2012;16(8):iii-iv, 1-76. doi: 10.3310/hta16080. |
| HTA20 | Critchley HOD, Warner P, Lee AJ, Brechin S, Guise J, et al. Evaluation of abnormal uterine bleeding: comparison of three outpatient procedures within cohorts defined by age and menopausal status. Health Technol Assess. 2004 Sep;8(34):iii-iv, 1-139. |
| HTA21 | Cross J, Elender F, Barton G, Clark A, Shepstone L, et al. A randomised controlled equivalence trial to determine the effectiveness and cost–utility of manual chest physiotherapy techniques in the management of exacerbations of chronic obstructive pulmonary disease (MATREX). Health Technol Assess. 2010 May;14(23):1-147, iii-iv. doi: 10.3310/hta14230. |
| HTA22 | Dennis M, Lewis S, Cranswick G Forbes J, FOOD Trial Collaboration. FOOD: a multicentre randomised trial evaluating feeding policies in patients admitted to hospital with a recent stroke. Health Technol Assess. 2006 Jan;10(2):iii-iv, ix-x, 1-120. |
| HTA23 | Donovon J, Hamdy F, Neal D, Peters T, Oliver S, et al. Prostate Testing for Cancer and Treatment (ProtecT) feasibility study. Health Technol Assess. 2003;7(14):1-88. |
| HTA24 | Dormandy E, Bryan S, Gulliford MC, Roberts TE, Ades AE, et al. Antenatal screening for haemoglobinopathies in primary care: a cohort study and cluster randomised trial to inform a simulation model. The Screening for Haemoglobinopathies in First Trimester (SHIFT) trial. Health Technol Assess. 2010 Apr;14(20):1-160. doi: 10.3310/hta14200. |
| HTA25 | Dumville JC, Worthy G, Soares PO, Bland JM, Cullum N, et al. VenUS II: a randomised controlled trial of larval therapy in the management of leg ulcers. Health Technol Assess. 2009 Nov;13(55):1-182, iii-iv. doi: 10.3310/hta13550. |
| HTA26 | Elliot RA, Payne K, Moore JK, Davies LM, Harper NJN. Which anaesthetic agents are costeffective in day surgery? Literature review, national survey of practice and randomised controlled trial. Health Technol Assess. 2002;6(30):1-264. |
| HTA27 | Epps H, Ginnelly L, Utley M, Southwood T, Gallivan S, et al. Is hydrotherapy cost-effective? A randomised controlled trial of combined hydrotherapy programmes compared with physiotherapy land techniques in children with juvenile idiopathic arthritis. Health Technol Assess. 2005 Oct;9(39):iii-iv, ix-x, 1-59. |
| HTA28 | Fader M, Cottenden A, Getliffe K, Gage H, Clarke-O’Neill S, et al. Absorbent products for urinary/faecal incontinence: a comparative evaluation of key product designs. Health Technol Assess. 2008 Jul;12(29):iii-iv, ix-185. |
| HTA29 | Farmer AJ, Wade AN, French DP, Simon J, Yudkin P, et al. Blood glucose self-monitoring in type 2 diabetes: a randomised controlled trial. Health Technol Assess. 2009 Feb;13(15):iii-iv, ix-xi, 1-50. doi: 10.3310/hta13150. |
| HTA30 | Fowler C, McAllister W, Plail R, Karim O, Yang Q. Randomised evaluation of alternative electrocsurgical modalities to treat bladder outflow obstruction in men with benign prostatic hyperplasia. Health Technol Assess. 2005 Feb;9(4):iii-iv, 1-30. |
| HTA31 | Garry R, Fountain J, Brown J, Manca A, Mason S, et al. EVALUATE hysterectomy trial: a multicentre randomised trial comparing abdominal, vaginal and laparoscopic methods of hysterectomy. Health Technol Assess. 2004 Jun;8(26):1-154. |
| HTA32 | George S, Pockney P, Primrose J, Smith H, Little P, et al. A prospective randomised comparison of minor surgery in primary and secondary care. The MiSTIC trial. Health Technol Assess. 2008 May;12(23):iii-iv, ix-38. |
| HTA33 | Gilbert FJ, Grant AM, Gillan MGC, Vale L, Scott NW, et al. Does early magnetic resonance imaging influence management or improve outcome in patients referred to secondary care with low back pain? A pragmatic randomised controlled trial. Health Technol Assess. 2004 May;8(17):iii, 1-131. |
| HTA34 | Glazener C, Boachie C, Buckley B, Cochran C, Dorey G, et al. Conservative treatment for urinary incontinence in Men After Prostate Surgery (MAPS): two parallel randomised controlled trials. Health Technol Assess. 2011 Jun;15(24):1-290, iii-iv. doi: 10.3310/hta15240. |
| HTA35 | Goodacre S, Bradburn M, Fitzgerald P, Cross E, Collinson P, et al. The RATPAC (Randomised Assessment of Treatment using Panel Assay of Cardiac markers) trial: a randomised controlled trial of point-of-care cardiac markers in the emergency department. Health Technol Assess. 2011 May;15(23):iii-xi, 1-102. doi: 10.3310/hta15230. |
| HTA36 | Goodyer IM, Dubicka B, Wilkinson P, Kelvin R, Roberts C, et al. A randomised controlled trial of cognitive behaviour therapy in adolescents with major depression treated by selective serotonin reuptake inhibitors. The ADAPT trial. lth Technol Assess. 2008 May;12(14):iii-iv, ix-60. |
| HTA37 | Gowers SG, Clark AF, Roberts C, Byford S, Barrett B, et al. A randomised controlled multicentre trial of treatments for adolescent anorexia nervosa including assessment of cost-effectiveness and patient acceptability – the TOuCAN trial. Health Technol Assess. 2010 Mar;14(15):1-98. doi: 10.3310/hta14150. |
| HTA38 | Gray AJ, Goodacre S, Newby DE, Masson MA, Sampson F, et al. A multicentre randomised controlled trial of the use of continuous positive airway pressure and non-invasive positive pressure ventilation in the early treatment of patients presenting to the emergency department with severe acute cardiogenic pulmonary oedema: the 3CPO trial. Health Technol Assess. 2009 Jul;13(33):1-106. doi: 10.3310/hta13330. |
| HTA39 | Gregory J, Robling M, Bennert K, Channon S, Cohen D, et al. Development and evaluation by a cluster randomised trial of a psychosocial intervention in children and teenagers experiencing diabetes: the DEPICTED study. Health Technol Assess. 2011 Aug;15(29):1-202. doi: 10.3310/hta15290. |
| HTA40 | Harvey S, Stevens K, Harrison D, Young D, Brampton W, et al. An evaluation of the clinical and cost-effectiveness of pulmonary artery catheters in patient management in intensive care: a systematic review and a randomised controlled trial. Health Technol Assess. 2006 Aug;10(29):iii-iv, ix-xi, 1-133. |
| HTA41 | Hay AD, Redmond NM, Costelloe C, Montgomery AA, Fletcher M, et al. Paracetamol and ibuprofen for the treatment of fever in children: the PITCH randomised controlled trial. Health Technol Assess. 2009 May;13(27):iii-iv, ix-x, 1-163. doi: 10.3310/hta13270. |
| HTA42 | Hewison J, Nixon J, Fountain J, Cocks K, Jones C, et al. Amniocentesis results: investigation of anxiety. The ARIA trial. Health Technol Assess. 2006;10(50):1-226. |
| HTA43 | Hobbs FD, Fitzmaurice DA, Jowett S, Mant J, Murray E, et al. A randomised controlled trial and cost-effectiveness study of systematic screening (targeted and total population screening) versus routine practice for the detection of atrial fibrillation in people aged 65 and over. The SAFE study. Health Technol Assess. 2005 Oct;9(40):iii-iv, ix-x, 1-74. |
| HTA44 | Iglesias C, Nelson EA, Cullum NA, Torgerson DJ, VenUS Team. VenUS I: a randomised controlled trial of two types of bandage for treating venous leg ulcers. Health Technol Assess. 2004 Jul;8(29):iii, 1-105. |
| HTA45 | Isaacs AJ, Critchley JA, See Tai S, Buckingham K, Westley D, et al. Exercise Evaluation Randomised Trial (EXERT): a randomised trial comparing GP referral for leisure centre-based exercise, community-based walking and advice only. Health Technol Assess. 2007 Mar;11(10):1-165, iii-iv. |
| HTA46 | Ismail K, Maissi E, Thomas S, Chalder T, Schmidt U, et al. A randomised controlled trial of cognitive behaviour therapy and motivational interviewing for people with type 1 diabetes mellitus with persistent sub-optimal glycaemic control: A Diabetes and Psychological Therapies (ADaPT) study. Health Technol Assess. 2010 May;14(22):1-101, iii-iv. doi: 10.3310/hta14220. |
| HTA47 | Jeffcoate WJ, Price PE, Phillips CJ, Game FL, Mudge E, et al. Randomised controlled trial of the use of three dressing preparations in the management of chronic ulceration of the foot in diabetes. Health Technol Assess. 2009 Nov;13(54):1-86, iii-iv. doi: 10.3310/hta13540. |
| HTA48 | Jolly K, Taylor R, Lip GYH, Greenfield S, Raftery J, et al. The Birmingham Rehabilitation Uptake Maximisation Study (BRUM). Homebased compared with hospital-based cardiac rehabilitation in a multi-ethnic population: cost-effectiveness and patient adherence. Health Technol Assess. 2007 Sep;11(35):1-118. |
| HTA49 | Kalra L, Evans A, Perez I, Knapp M, Swift C, et al. A randomised controlled comparison of alternative strategies in stroke care. Health Technol Assess. 2005 May;9(18):iii-iv, 1-79. |
| HTA50 | Keating JF, Grant A, Masson M, Scott NW, Forbes JF. Displaced intracapsular hip fractures in fit, older people: a randomised comparison of reduction and fixation, bipolar hemiarthroplasty and total hip arthroplasty. Health Technol Assess. 2005 Oct;9(41):iii-iv, ix-x, 1-65. |
| HTA51 | Kendrick D, Fielding K, Bentley E, Miller P. The role of radiography in primary care patients with low back pain of at least 6 weeks duration: a randomised (unblinded) controlled trial. Health Technol Assess 2001;5(30). |
| HTA52 | Kendrick T, Chatwin J, Dowrick C, Tylee A, Morriss R, et al. Randomised controlled trial to determine the clinical effectiveness and cost-effectiveness of selective serotonin reuptake inhibitors plus supportive care, versus supportive care alone, for mild to moderate depression with somatic symptoms in primary care: the THREAD (THREshold for AntiDepressant response) study. Health Technol Assess. 2009 Apr;13(22):iii-iv, ix-xi, 1-159. doi: 10.3310/hta13220. |
| HTA53 | Kendrick T, Simons L, Mynors-Wallis L, Gray A, Lathlean J, et al. A trial of problem-solving by community mental health nurses for anxiety, depression and life difficulties among general practice patients. The CPN-GP study. Health Technol Assess. 2005 Sep;9(37):1-104, iii. |
| HTA54 | Kennedy A, Robinson A, Nelson E, Rogers A, Reeves D, et al. A randomised controlled trial to assess the impact of a package comprising a patient-orientated, evidence-based self-help guidebook and patient-centred consultations on disease management and satisfaction in inflammatory bowel disease. Health Technol Assess. 2003;7(28):iii, 1-113. |
| HTA55 | Kennedy ADM, Sculpher MJ, Coulter A, Dwyer N, Rees M. A multicentre randomised controlled trial assessing the costs and benefits of using structured information and analysis of women’s preferences in the management of menorrhagia. Health Technol Assess. 2003;7(8):1-76. |
| HTA56 | Kennedy TM, Chalder T, McCrone P, Darnley S, Knapp M, et al. Cognitive behavioural therapy in addition to antispasmodic therapy for irritable bowel syndrome in primary care: randomised controlled trial. Health Technol Assess. 2006 Jun;10(19):iii-iv, ix-x, 1-67. |
| HTA57 | Kerry S, Hilton S, Patel S, Dundas D. Routine referral for radiography of patients presenting with low back pain: is patients’ outcome influenced by GPs’ referral for plain radiography? Health Technol Assess. 2000;4(20):i-iv, 1-119. |
| HTA58 | King M, Sibbald B, Ward E. Randomised controlled trial of non-directive counselling, cognitive– behaviour therapy and usual general practitioner care in the management of depression as well as mixed anxiety and depression in primary care. Health Technol Assess. 2000;4(19):1-83. |
| HTA59 | Kinley H, Czoski-Murray C, George S, McCabe C. Extended scope of nursing practice: a multicentre randomised controlled trial of appropriately trained nurses and pre-registration house officers in pre-operative assessment in elective general surgery. Health Technol Assess. 2001;5(20):1-87. |
| HTA60 | Kitchener HC, Almonte M, Gilham C, Dowie R, Stoykova B, et al. ARTISTIC: a randomised trial of human papillomavirus (HPV) testing in primary cervical screening. Health Technol Assess. 2009 Nov;13(51):1-150, iii-iv. doi: 10.3310/hta13510. |
| HTA61 | Kitchener HC, Blanks R, Cubie H, Desai M, Dunn G, et al. MAVARIC – a comparison of automation-assisted and manual cervical screening: a randomised controlled trial. Health Technol Assess. 2011 Jan;15(3):iii-iv, ix-xi, 1-170. doi: 10.3310/hta15030. |
| HTA62 | Lamb SE, Lall R, Hansen Z, Castelnuovo E, Withers EJ, et al. A multicentred randomised controlled trial of a primary care-based cognitive behavioural programme for low back pain. The Back Skills Training (BeST) trial. Health Technol Assess. 2010 Aug;14(41):1-253, iii-iv. doi: 10.3310/hta14410. |
| HTA63 | Lamb SE, Williams MA, Williamson EM, Gates S, Withers EJ, et al. Managing Injuries of the Neck Trial (MINT): a randomised controlled trial of treatments for whiplash injuries. Health Technol Assess. 2012;16(49):iii-iv, 1-141. doi: 10.3310/hta16490. |
| HTA64 | Lenney W, McKay AJ, Tudur Smith C, Williamson PR, James M et al. Management of Asthma in School age Children On Therapy (MASCOT): a randomised, double-blind, placebo-controlled, parallel study of efficacy and safety. Health Technol Assess. 2013 Feb;17(4):1-218. doi: 10.3310/hta17040. |
| HTA65 | Lewis SW, Davies L, Jones PB, Barnes TRE, Murray RM, et al. Randomised controlled trials of conventional antipsychotic versus new atypical drugs, and new atypical drugs versus clozapine, in people with schizophrenia responding poorly to, or intolerant of, current drug treatment. Health Technol Assess. 2006 May;10(17):iii-iv, ix-xi, 1-165. |
| HTA66 | Little P, Turner S, Rumsby K, Warner G, Moore M, et al. Dipsticks and diagnostic algorithms in urinary tract infection: development and validation, randomised trial, economic analysis, observational cohort and qualitative study. Health Technol Assess. 2009 Mar;13(19):iii-iv, ix-xi, 1-73. doi: 10.3310/hta13190. |
| HTA67 | Lock C, Wilson J, Steen N, Eccles M, Mason H, et al. North of England and Scotland Study of Tonsillectomy and Adeno-tonsillectomy in Children (NESSTAC): a pragmatic randomised controlled trial with a parallel nonrandomised preference study. Health Technol Assess. 2010 Mar;14(13):1-164, iii-iv. doi: 10.3310/hta14130. |
| HTA68 | Lord J, Victor C, Littlejohns P, Ross FM. Economic evaluation of a primary care-based education programme for patients with osteoarthritis of the knee. Health Technol Assess. 1999;3(23):1-55. |
| HTA69 | Low N, McCarthy A, Macleod J, Salisbury C, Campbell R, et al. Epidemiological, social, diagnostic and economic evaluation of population screening for genital chlamydial infection. Health Technol Assess. 2007 Mar;11(8):iii-iv, ix-xii, 1-165. |
| HTA70 | MacArthur C, Winter HR, Bick DE, Lilford RJ, Lancashire RJ, et al. Redesigning postnatal care: a randomised controlled trial of protocol-based midwifery-led care focused on individual women’s physical and psychological health needs. Health Technol Assess. 2003;7(37):1-98. |
| HTA71 | Marson AG, Appleton R, Baker GA, Chadwick DW, Doughty J, et al. A randomised controlled trial examining the longer-term outcomes of standard versus new antiepileptic drugs. The SANAD trial. Health Technol Assess. 2007 Oct;11(37):iii-iv, ix-x, 1-134. |
| HTA72 | McCarthy CJ, Mills PM, Pullen R, Richardson G, Hawkins N, et al. Supplementation of a home-based exercise programme with a class-based programme for people with osteoarthritis of the knees: a randomised controlled trial and health economic analysis. Health Technol Assess. 2004 Nov;8(46):iii-iv, 1-61. |
| HTA73 | McLoughlin DM, Mogg A, Eranti S, Pluck G, Purvis R, et al. The clinical effectiveness and cost of repetitive transcranial magnetic stimulation versus electroconvulsive therapy in severe depression: a multicentre pragmatic randomised controlled trial and economic analysis. Health Technol Assess. 2007 Jul;11(24):1-54. |
| HTA74 | Michaels JA, Campbell WB, King BM, MacIntyre J, Palfreyman SJ, et al. A prospective randomised controlled trial and economic modelling of antimicrobial silver dressings versus non-adherent control dressings for venous leg ulcers: the VULCAN trial. Health Technol Assess. 2009 Nov;13(56):1-114, iii. doi: 10.3310/hta13560. |
| HTA75 | Mihaylov S, Stark C, McColl E, Steen N, Vanoli A, et al. Stepped treatment of older adults on laxatives. The STOOL trial. Health Technol Assess. 2008 May;12(13):iii-iv, ix-139. |
| HTA76 | Morgan K, Dixon S, Mathers N, Thompson J, Tomeny M. Psychological treatment for insomnia in the regulation of long-term hypnotic drug use. Health Technol Assess. 2004 Feb;8(8):iii-iv, 1-68. |
| HTA77 | Morrell CJ, Piby S, Stewart P. Costs and benefits of community postnatal support workers: a randomised controlled trial. Health Technol Assess. 2000;4(6):1-100. |
| HTA78 | Morrell CJ, Warner R, Slade P, Dixon S, Walters S, et al. Psychological interventions for postnatal depression: cluster randomised trial and economic evaluation. The PoNDER trial. Health Technol Assess. 2009 Jun;13(30):iii-iv, xi-xiii, 1-153. doi: 10.3310/hta13300. |
| HTA79 | Newman SP, Cooke D, Casbard A, Walker S, Meredith S, et al. A randomised controlled trial to compare minimally invasive glucose monitoring devices with conventional monitoring in the management of insulin-treated diabetes mellitus (MITRE). Health Technol Assess. 2009 May;13(28):iii-iv, ix-xi, 1-194. doi: 10.3310/hta13280. |
| HTA80 | Nixon J, Nelson EA, Cranny G, Iglesias CP, Hawkins K, et al. Pressure relieving support surfaces: a randomised evaluation. Health Technol Assess. 2006 Jul;10(22):iii-iv, ix-x, 1-163. |
| HTA81 | O’Dowd H, Gladwell P, Rogers CA, Hollinghurst S, Gregory A. Cognitive behavioural therapy in chronic fatigue syndrome: a randomised controlled trial of an outpatient group programme. Health Technol Assess. 2006 Oct;10(37):iii-iv, ix-x, 1-121. |
| HTA82 | Ozolins M, Eady EA, Avery A, Cunliffe WJ, O’Neill C, et al. Randomised controlled multiple treatment comparison to provide a cost-effectiveness rationale for the selection of antimicrobial therapy in acne. Health Technol Assess. 2005 Jan;9(1):iii-212. |
| HTA83 | Parker SG, Oliver P, Pennington M, Bond J, Jagger C, et al. Rehabilitation of older patients: day hospital compared with rehabilitation at home. A randomised controlled trial. Health Technol Assess. 2009 Aug;13(39):1-143, iii-iv. doi: 10.3310/hta13390. |
| HTA84 | Peek GJ, Elbourne D, Mugford M, Tiruvoipati R, Wilson A, et al. Randomised controlled trial and parallel economic evaluation of conventional ventilatory support versus extracorporeal membrane oxygenation for severe adult respiratory failure (CESAR). Health Technol Assess. 2010 Jul;14(35):1-46. doi: 10.3310/hta14350. |
| HTA85 | Perel P, Al-Shahi Salman R, Kawahara T, Morris Z, Prieto-Merino D, et al. CRASH-2 (Clinical Randomisation of an Antifibrinolytic in Significant Haemorrhage) intracranial bleeding study: the effect of tranexamic acid in traumatic brain injury – a nested, randomised, placebo-controlled trial. Health Technol Assess. 2012;16(13):iii-xii, 1-54. doi: 10.3310/hta16130. |
| HTA86 | Peveler R, Kendrick T, Buxton M, Longworth L, Baldwin D, et al. A randomised controlled trial to compare the cost-effectiveness of tricyclic antidepressants, selective serotonin reuptake inhibitors and lofepramine. Health Technol Assess. 2005 May;9(16):1-134, iii. |
| HTA87 | Pickard R, Lam T, MacLennan G, Starr K, Kilonzo M, et al. Types of urethral catheter for reducing symptomatic urinary tract infections in hospitalised adults requiring short-term catheterisation: multicentre randomised controlled trial and economic evaluation of antimicrobial- and antisepticimpregnated urethral catheters (the CATHETER trial). Health Technol Assess. 2012 Nov;16(47):1-197. doi: 10.3310/hta16470. |
| HTA88 | Potter J, Mistri A, Brodie F, Chernova J, Wilson E, et al. Controlling Hypertension and Hypotension Immediately Post Stroke (CHHIPS) – a randomised controlled trial. Health Technol Assess. 2009 Jan;13(9):iii, ix-xi, 1-73. doi: 10.3310/hta13090. |
| HTA89 | Prescott RJ, Kunkler IH, Williams LJ, King CC, Jack W, et al. A randomised controlled trial of postoperative radiotherapy following breast-conserving surgery in a minimum-risk older population. The PRIME trial. Health Technol Assess. 2007 Aug;11(31):1-149, iii-iv. |
| HTA90 | Price C, Arden N, Coglan L, Rogers P. Cost-effectiveness and safety of epidural steroids in the management of sciatica. Health Technol Assess. 2005 Aug;9(33):1-58, iii. |
| HTA91 | Price D, Musgrave S, Wilson E, Sims E, Shepstone L, et al. A pragmatic single-blind randomised controlled trial and economic evaluation of the use of leukotriene receptor antagonists in primary care at steps 2 and 3 of the national asthma guidelines (ELEVATE study). Health Technol Assess. 2011 May;15(21):1-132. doi: 10.3310/hta15210. |
| HTA92 | Reeves BC, Angelini GD, Bryan AJ, Taylor FC, Cripps T, et al. A multi-centre randomised controlled trial of minimally invasive direct coronary bypass grafting versus percutaneous transluminal coronary angioplasty with stenting for proximal stenosis of the left anterior descending coronary artery. Health Technol Assess. 2004 Apr;8(16):1-43. |
| HTA93 | Roberts I, Shakur H, Coats I, Hunt B, Balogun E, et al.The CRASH-2 trial: a randomised controlled trial and economic evaluation of the effects of tranexamic acid on death, vascular occlusive events and transfusion requirement in bleeding trauma patients. Health Technol Assess. 2013 Mar;17(10):1-79. doi: 10.3310/hta17100. |
| HTA94 | Robson SC, Kelly T, Howel D, Deverill M, Hewison J, et al. Randomised preference trial of medical versus surgical termination of pregnancy less than 14 weeks’ gestation (TOPS). Health Technol Assess. 2009 Nov;13(53):1-124, iii-iv. doi: 10.3310/hta13530. |
| HTA95 | Scawn N, Saul D, Pathak D, Matata B, Kemp I, et al. A pilot randomised controlled trial in intensive care patients comparing 7 days’ treatment with empirical antibiotics with 2 days’ treatment for hospital-acquired infection of unknown origin. Health Technol Assess. 2012 Sep;16(36):i-xiii, 1-70. |
| HTA96 | Sharp DJ, Chew-Graham CA, Tylee A, Lewis G, Howard L, et al. A pragmatic randomised controlled trial to compare antidepressants with a community-based psychosocial intervention for the treatment of women with postnatal depression: the RESPOND trial. Health Technol Assess. 2010 Sep;14(43):iii-iv, ix-xi, 1-153. doi: 10.3310/hta14430. |
| HTA97 | Sharples L, Hughes V, Crean A, Dyer M, Buxton M, et al. Cost-effectiveness of functional cardiac testing in the diagnosis and management of coronary artery disease: a randomised controlled trial. The CECaT trial. Health Technol Assess. 2007; 11(49): 1-136 |
| HTA98 | Sharples LD, Jackson C, Wheaton E, Griffith G, Annema JT, et al. Clinical effectiveness and cost effectiveness of endobronchial and endoscopic ultrasound relative to surgical staging in potentially resectable lung cancer: results from the ASTER randomised controlled trial. Health Technol Assess. 2012;16(18):1-75, iii-iv. doi: 10.3310/hta16180. |
| HTA99 | Shaw L, Rodgers H, Price C, van Wijck F, Shackley P, et al. BoTULS: a multicentre randomised controlled trial to evaluate the clinical effectiveness and cost-effectiveness of treating upper limb spasticity due to stroke with botulinum toxin type A. Health Technol Assess. 2010 May;14(26):1-113, iii-iv. doi: 10.3310/hta14260. |
| HTA100 | Shenfine J, McNamee P, Steen N, Bond J, Griffin SM. A pragmatic randomised controlled trial of the cost-effectiveness of palliative therapies for patients with inoperable oesophageal cancer. Health Technol Assess. 2005 Feb;9(5):iii, 1-121. |
| HTA101 | Simpson S, Corney R, Fitzgerald P, Beecham J. A randomised controlled trial to evaluate the effectiveness and cost-effectiveness of counselling patients with chronic depression. Health Technol Assess. 2000;4(36):1-83. |
| HTA102 | Simpson WM, Johnstone FD, Boyd FM. A randomised controlled trial of different approaches to universal antenatal HIV testing: uptake and acceptability and Annex: Antenatal HIV testing – assessment of a routine voluntary approach. Health Technol Assess. 1999;3(4):1-112. |
| HTA103 | Speed C, Heaven B, Adamson A, Bond J, Corbett S, et al. LIFELAX – diet and LIFEstyle versus LAXatives in the management of chronic constipation in older people: randomised controlled trial. Health Technol Assess. 2010 Nov;14(52):1-251. doi: 10.3310/hta14520. |
| HTA104 | Sullivan FM, Swan IRC, Donnan PT, Morrison JM, Smith BH, et al. A randomised controlled trial of the use of aciclovir and/or prednisolone for the early treatment of Bell’s palsy: the BELLS study. Health Technol Assess. 2009 Oct;13(47):iii-iv, ix-xi 1-130. doi: 10.3310/hta13470. |
| HTA105 | Suri R, Wallis C, Bush A, Thompson S. A comparative study of hypertonic saline, daily and alternate-day rhDNase in children with cystic fibrosis. Health Technol Assess. 2002;6(34):iii, 1-60. |
| HTA106 | Symmons D, Tricker K, Roberts C, Davies L, Dawes P, et al. The British Rheumatoid Outcome Study Group (BROSG) randomised controlled trial to compare the effectiveness and cost-effectiveness of aggressive versus symptomatic therapy in established rheumatoid arthritis. Health Technol Assess. 2005 Sep;9(34):iii-iv, ix-x, 1-78 |
| HTA107 | Taylor P, Champness J, Given-Wilson R, Johnston K, Potts H. Impact of computer-aided detection prompts on the sensitivity and specificity of screening mammography. Health Technol Assess. 2005 Feb;9(6):iii, 1-58. |
| HTA108 | Thomas KJ, MacPherson H, Ratcliffe J, Thorpe L, Brazier J, et al. Longer term clinical and economic benefits of offering acupuncture care to patients with chronic low back pain. Health Technol Assess. 2005 Aug;9(32):iii-iv, ix-x, 1-109. |
| HTA109 | Thomas KS, Koller K, Dean T, O’Leary CJ, Sach TH, et al. A multicentre randomised controlled trial and economic evaluation of ion-exchange water softeners for the treatment of eczema in children: the Softened Water Eczema Trial (SWET). Health Technol Assess. 2011 Feb;15(8):v-vi, 1-156. doi: 10.3310/hta15080. |
| HTA110 | Townsend J, Wolke D, Hayes J, Davé S, Rogers C, et al. Routine examination of the newborn: the EMREN study. Evaluation of an extension of the midwife role including a randomised controlled trial of appropriately trained midwives and paediatric senior house officers. Health Technol Assess. 2004 Apr;8(14):iii-iv, ix-xi, 1-100. |
| HTA111 | Turnbull LW, Brown SR, Olivier C, Harvey I, Brown J, et al. Multicentre randomised controlled trial examining the cost-effectiveness of contrast-enhanced high field magnetic resonance imaging in women with primary breast cancer scheduled for wide local excision (COMICE). Health Technol Assess. 2010 Jan;14(1):1-182. doi: 10.3310/hta14010. |
| HTA112 | Turner J, Nicholl J, Webber L, Cox H. A randomised controlled trial of prehospital intravenous fluid replacement therapy in serious trauma. Health Technol Assess. 2000;4(31):1-57. |
| HTA113 | Tyrer P, Oliver-Africano P, Romeo R, Knapp M, Dickens S, et al. Neuroleptics in the treatment of aggressive challenging behaviour for people with intellectual disabilities: a randomised controlled trial (NACHBID). Health Technol Assess. 2009 Apr;13(21):iii-iv, ix-xi, 1-54. doi: 10.3310/hta13210. |
| HTA114 | Underwood M, Ashby D, Carnes D, Castelnuovo E, Cross P, et al. Topical or oral ibuprofen for chronic knee pain in older people. The TOIB study. Health Technol Assess. 2008 May;12(22):iii-iv, ix-155. |
| HTA115 | Vickers AJ, Rees RW, Zollman CE, McCarney R, Smith CM, et al. Acupuncture of chronic headache disorders in primary care: randomised controlled trial and economic analysis. Health Technol Assess. 2004 Nov;8(48):iii, 1-35. |
| HTA116 | Wallace P, Barber J, Clayton W, Currell R, Fleming K, et al. Virtual outreach: a randomised controlled trial and economic evaluation of joint teleconferenced medical consultations. Health Technol Assess. 2004 Dec;8(50):1-106, iii-iv. |
| HTA117 | Waterhouse JC, Walters SJ, Oluboyede Y, Lawson RA. A randomised 2 × 2 trial of community versus hospital pulmonary rehabilitation for chronic obstructive pulmonary disease followed by telephone or conventional follow-up. Health Technol Assess. 2010 Feb;14(6):i-v, vii-xi, 1-140. doi: 10.3310/hta14060. |
| HTA118 | Watson JM, Kang’ombe AR, Soares MO, Chuang L-H, Worthy G, et al. VenUS III: a randomised controlled trial of therapeutic ultrasound in the management of venous leg ulcers. Health Technol Assess. 2011 Mar;15(13):1-192. doi: 10.3310/hta15130. |
| HTA119 | Weindling AM, Cunningham CC, Glenn SM, Edwards RT, Reeves DJ. Additional therapy for young children with spastic cerebral palsy: a randomised controlled trial. Health Technol Assess. 2007 May;11(16):iii-iv, ix-x, 1-71. |
| HTA120 | Wiggins M, Oakley A, Roberts I, Turner H, Rajan L, et al. The Social Support and Family Health Study: a randomised controlled trial and economic evaluation of two alternative forms of postnatal support for mothers living in disadvantaged inner-city areas. Health Technol Assess. 2004 Sep;8(32). doi: 10.3310/hta8320 |
| HTA121 | Williams J, Russell I, Durai D, Cheung W-Y, Farrin A, et al. What are the clinical outcome and cost-effectiveness of endoscopy undertaken by nurses when compared with doctors? A Multi-Institution Nurse Endoscopy Trial (MINuET). Health Technol Assess. 2006 Oct;10(40):iii-iv, ix-x, 1-195. |
| HTA122 | Williams LJ, Kunkler IH, King CC, Jack W, van der Pol M. A randomised controlled trial of post-operative radiotherapy following breast-conserving surgery in a minimum-risk population. Quality of life at 5 years in the PRIME trial. Health Technol Assess. 2011 Mar;15(12):i-xi, 1-57. doi: 10.3310/hta15120. |
| HTA123 | Williamson I, Benge S, Barton S, Petrou S, Letley L, et al. A double-blind randomised placebo controlled trial of topical intranasal corticosteroids in 4- to 11-year-old children with persistent bilateral otitis media with effusion in primary care. Health Technol Assess. 2009 Aug;13(37). doi: 10.3310/hta13370. |
| HTA124 | Wilson BJ, Torrance N, Mollison J, Wordsworth S, Gray JR, et al. Improving the referral process for familial breast cancer genetic counselling: findings of three randomised controlled trials of two interventions. Health Technol Assess. 2005 Feb;9(3):iii-iv, 1-126. |
| HTA125 | Woods RT, Bruce E, Edwards RT, Elvish R, Hoare Z, et al. REMCARE: reminiscence groups for people with dementia and their family caregivers - effectiveness and cost-effectiveness pragmatic multicentre randomised trial. Health Technol Assess. 2012;16(48):v-xv, 1-116. doi: 10.3310/hta16480. |
| HTA126 | Wright M, Grieve R, Roberts J, Main J, Thomas HC, et al. Health benefits of antiviral therapy for mild chronic hepatitis C: randomised controlled trial and economic evaluation. Health Technol Assess. 2006 Jul;10(21):1-113, iii. |
| HTA127 | Zermansky AG, Petty DR, Raynor DK, Lowe CJ, Freemantle N, et al. Clinical medication review by a pharmacist of patients on repeat prescriptions in general practice: a randomised controlled trial. Health Technol Assess. 2002;6(20):1-86. |
| Lancet1 | Atagi S, Kawahara M, Yokoyama A, Okamoto H, Yamamoto N, et al. Thoracic radiotherapy with or without daily low-dose carboplatin in elderly patients with non-small-cell lung canceer: a randomised, controlled, phase 3 trial by the Japan Clinical Oncology Group (JC0G0301). Lancet Oncol. 2012 Jul;13(7):671-8. doi: 10.1016/S1470-2045(12)70139-0. Epub 2012 May 22. |
| Lancet2 | Baulac M, Brodie MJ, Patten A, Segieth J, Giorgi L. Efficacy and tolerability of zonisamide versus controlled-release carbamazepine for newly diagnosed partial epilepsy: a phase 3, randomised, double-blind, non-inferiority trial. Lancet Neurol. 2012 Jul;11(7):579-88. doi: 10.1016/S1474-4422(12)70105-9. Epub 2012 Jun 8. |
| Lancet3 | Biondi A, Schrappe M, De Lorenzo P, Castor A, Lucchini G, et al. Imatinib after induction for treatment of children and adolescents with Philadelphia-chromosome-positive acute lymphoblastic leukaemia (EsPhALL): a randomised, open-label, intergroup study. Lancet Oncol. 2012 Sep;13(9):936-45. doi: 10.1016/S1470-2045(12)70377-7. Epub 2012 Aug 14. |
| Lancet4 | Bolla M, van Poppel H, Tombal B, Vekemans K, Da Pozzo L, et al. Postoperative radiotherapy after radical prostatectomy for high-risk prostate cancer: long-term results of a randomised controlled trial (EORTC trial 22911). Lancet. 2012 Dec 8;380(9858):2018-27. doi: 10.1016/S0140-6736(12)61253-7. Epub 2012 Oct 19. |
| Lancet5 | Bornhäuser M, Kienast J, Trenschel R, Burchert A, Hegenbart U, et al. Reduced-intensity conditioning versus standard conditioning before allogeneic haemopoietic cell transplantation in patients with acute myeloid leukaemia in first complete remission: a prospective, open-label randomised phase 3 trial. Lancet Oncol. 2012 Oct;13(10):1035-44. doi: 10.1016/S1470-2045(12)70349-2. Epub 2012 Sep 7. |
| Lancet6 | Camenzind E, Wijns W, Mauri L, Kurowski V, Parikh K, et al. Stent thrombosis and major clinical events at 3 years after zotarolimus-eluting or sirolimus-eluting coronary stent implantation: a randomised, multicentre, open-label, controlled trial. Lancet. 2012 Oct 20;380(9851):1396-405. doi: 10.1016/S0140-6736(12)61336-1. Epub 2012 Aug 27. |
| Lancet7 | Cohen JA, Coles AJ, Arnold DL, Confavreux C, Fox EJ, et al. Alemtuzumab versus interferon beta 1a as first-line treatment for patients with relapsing-remitting multiple sclerosis: a randomised controlled phase 3 trial. Lancet. 2012 Nov 24;380(9856):1819-28. doi: 10.1016/S0140-6736(12)61769-3. Epub 2012 Nov 1. |
| Lancet8 | Coles AJ, Twyman CL, Arnold DL, Cohen JA, Confavreux C, et al. Alemtuzumab for patients with relapsing multiple sclerosis after disease-modifying therapy: a randomised controlled phase 3 trial. Lancet. 2012 Nov 24;380(9856):1829-39. doi: 10.1016/S0140-6736(12)61768-1. Epub 2012 Nov 1. |
| Lancet9 | Corbel V, Akogbeto M, Damien GB, Djenontin A, Chandre F, et al. Combination of malaria vector control interventions in pyrthroid resistance area in Benin: a cluster randomised controlled trial. Lancet Infect Dis. 2012 Aug;12(8):617-26. doi: 10.1016/S1473-3099(12)70081-6. Epub 2012 Jun 7. |
| Lancet10 | Dávalos A, Alvarez-Sabín J, Castillo J, Díez-Tejedor E, Ferro J, et al. Citicoline in the treatment of acute ischaemic stroke: an international, randomised, multicentre, placebo-controlled study (ICTUS trial). Lancet. 2012 Jul 28;380(9839):349-57. doi: 10.1016/S0140-6736(12)60813-7. Epub 2012 Jun 11. |
| Lancet11 | DeJesus E, Rockstroh JK, Henry K, Molina J-M, Gathe J, et al. Co-formulated elvitegravir, cobicistat, emtricitabine, and tenofovir disoproxil fumerate versus ritonavir-boosted atazanavir plus co-formulated emtricitabine and tenofovir disoproxil fumarate for initial treatment of HIV-1 infection: a randomised, double-blind, phase 3, non-inferiority trial. Lancet. 2012 Jun 30;379(9835):2429-38. doi: 10.1016/S0140-6736(12)60918-0. |
| Lancet12 | Diacon AH, Dawson R, von Groote-Bidlingmaier F, Symons G, Venter A, et al. 14-day bactericidal activity of PA-824, bedaquiline, pyrazinamide, and moxifloxacin combinations: a randomised trial. Lancet. 2012 Sep 15;380(9846):986-93. doi: 10.1016/S0140-6736(12)61080-0. Epub 2012 Jul 23. |
| Lancet13 | Fairall L, Bachmann MO, Lombard C, Timmerman V, Uebel K, et al. Task shifting of antiretroviral treatment from doctors to primary-care nurses in South Africa (STRETCH): a pragmatic, parallel, cluster-randomised trial. Lancet. 2012 Sep 8;380(9845):889-98. doi: 10.1016/S0140-6736(12)60730-2. Epub 2012 Aug 15. |
| Lancet14 | Fizazi K, Scher HI, Molina A, Logothetis CJ, Chi KN, et al. Abiraterone acetate for treatment of metastatic castration-resistant prostate cancer: final overall survival analysis of the COU-AA-301 randomised, double-blind, placebo-controlled phase 3 study. Lancet Oncol. 2012 Oct;13(10):983-92. doi: 10.1016/S1470-2045(12)70379-0. Epub 2012 Sep 18. |
| Lancet15 | FOxTROT Collaborative Group. Feasibility of preoperative chemotherapy for locally advanced, operable colon cancer: the pilot phase of a randomised controlled trial. Lancet Oncol. 2012 Nov;13(11):1152-60. doi: 10.1016/S1470-2045(12)70348-0. Epub 2012 Sep 25. |
| Lancet16 | Gallwitz B, Rosenstock J, Rauch T, Bhattacharya S, Patel S, et al. 2-year efficacy and safety of linagliptin compared with glimepiride in patients with type 2 diabetes inadequately controlled on metformin: a randomised, double-blind, non-inferiority trial. Lancet. 2012 Aug 4;380(9840):475-83. doi: 10.1016/S0140-6736(12)60691-6. Epub 2012 Jun 28. |
| Lancet17 | Giugliano RP, Desai NR, Kohli P, Rogers WJ, Somaratne R, et al. Efficacy, safety, and tolerability of a monoclonal antibody to proprotein convertase subtilisin/kexin type 9 in combination with a statin in patients with hypercholesterolaemia (LAPLACE-TIMI 57): a randomised, placebo-controlled, dose-ranging, phase 2 study. Lancet. 2012 Dec 8;380(9858):2007-17. doi: 10.1016/S0140-6736(12)61770-X. Epub 2012 Nov 6. |
| Lancet18 | Gramont A de, Cutsem EV, Schmoll H-J, Tabernero J, Clarke S, et al. Bevacizumab plus oxaliplatin-based chemotherapy as adjuvant treatment for colon cancer (AVANT): a phase 3 randomised controlled trial. Lancet Oncol. 2012 Dec;13(12):1225-33. doi: 10.1016/S1470-2045(12)70509-0. Epub 2012 Nov 16. |
| Lancet19 | Hauschild A, Grob JJ, Demidov LV, Jouary T, Gutzmer R, et al. Dabrafenib in BRAF-mutated metastatic melanoma: a multicentre, open-label, phase 3 randomised controlled trial. Lancet. 2012 Jul 28;380(9839):358-65. doi: 10.1016/S0140-6736(12)60868-X. Epub 2012 Jun 25. |
| Lancet20 | Hill MD, Martin RH, Mikulis D, Wong JH, Silver FL, et al. Safety and efficacy of NA-1 in patients with iatrogenic stroke after endovascular aneurysm repair (ENACT): a phase 2, randomised, double-blind, placebo-controlled trial. Lancet Neurol. 2012 Nov;11(11):942-50. doi: 10.1016/S1474-4422(12)70225-9. Epub 2012 Oct 8. |
| Lancet21 | Hong YS, Park YS, Lim HY, Lee J, Kim TW, et al. S-1 plus oxaliplatin versus capecitabine plus oxaliplatin for first-line treatment of patients with metastatic colorectal cancer: a randomised, non-inferiority phase 3 trial. Lancet Oncol. 2012 Nov;13(11):1125-32. doi: 10.1016/S1470-2045(12)70363-7. Epub 2012 Oct 10. |
| Lancet22 | Ismael G, Hegg R, Muehibauer S, Heinzmann D, Lum B, et al. Subcutaneous versus intravenous administration of (neo)adjuvant trastuzumab in patients with HER2-positive, clinical stage I–III breast cancer (HannaH study): a phase 3, open-label, multicentre, randomised trial. Lancet Oncol. 2012 Sep;13(9):869-78. doi: 10.1016/S1470-2045(12)70329-7. Epub 2012 Aug 9. |
| Lancet23 | Jamieson DJ, Chasela CS, Hudgens MG, King CC, Jourtis AP, et al. Maternal and infant antiretrovial regimens to orevent postnatal HIV-1 transmission: 48-week follow-up of the BAN randomised controlled trial. Lancet. 2012 June 30; 379(9835): 2449–2458. doi:10.1016/S0140-6736(12)60321-3. |
| Lancet24 | Kantarjian H, Faderl S, Garcia-Manero G, Luger S, Venugopal P, et al. Oral sapacitabine for the treatment of acute myeloid leukaemia in elderly patients: a randomised phase 2 study. Lancet Oncol. 2012 Nov;13(11):1096-104. doi: 10.1016/S1470-2045(12)70436-9. Epub 2012 Oct 15. |
| Lancet25 | Kirchhof P, Andresen D, Bosch R, Borggrefe M, Meinertz T, et al. Short-term versus long-term antiarrhythmic drug treatment after cardioversion of atrial fibrillation (Flex-SL): a prospective, randomised, open-label, blinded endpoint assessment trial. Lancet. 2012 Jul 21;380(9838):238-46. doi: 10.1016/S0140-6736(12)60570-4. Epub 2012 Jun 18. |
| Lancet26 | Kirsner RS, Marston WA, Snyder RJ, Lee TD, Innes Cargill D, et al. Spray-applied cell therapy with human allogeneic fibroblasts and keratinocytes for the treatment of chronic venous leg ulcers: a phase 2, multicentre, double-blind, randomised, placebo-controlled trial. Lancet. 2012 Sept 15;380(9846):977-85. doi: 10.1016/S0140-6736(12)60644-8. |
| Lancet27 | Koren MJ, Scott R, Kim JB, Knusel B, Liu T, et al. Efficacy, safety, and tolerability of a monoclonal antibody to proprotein convertase subtilisin/kexin type 9 as monotherapy in patients with hypercholesterolaemia (MENDEL): a randomised, double-blind, placebo-controlled, phase 2 study. Lancet. 2012 Dec 8;380(9858):1995-2006. doi: 10.1016/S0140-6736(12)61771-1. Epub 2012 Nov 6. |
| Lancet28 | Kuhn L, Coovadia A, Strehlau R, Martens L, Hu CC, et al. Switching children previously exposed to neviropine to nevirapine-based treatment after initial suppression with a protease-inhibitor-based regimen: long-term follow-up of a randomised, open-label trial. Lancet Infect Dis. 2012 Jul;12(7):521-30. doi: 10.1016/S1473-3099(12)70051-8. Epub 2012 Mar 16. |
| Lancet29 | Laharie D, Bourreille A, Branche J, Allez M, Bouhnik Y, et al. Ciclosporin versus infliximab in patients with severe ulcerative colitis refractory to intravenous steroids: a parallel, open-label randomised controlled trial. Lancet. 2012 Dec 1;380(9857):1909-15. doi: 10.1016/S0140-6736(12)61084-8. Epub 2012 Oct 10. |
| Lancet30 | Leboulleux S, Bastholt L, Krause T, de la Fouchardiere C, Tennvall J, et al. Vandetanib in locally advanced or metastatic differentiated thyroid cancer: a randomised, double-blind, phase 2 trial. Lancet Oncol. 2012 Sep;13(9):897-905. doi: 10.1016/S1470-2045(12)70335-2. Epub 2012 Aug 14. |
| Lancet31 | Lee SM, Khan I, Upadhyay S, Lewanski C, Falk S, et al. First-line erlotinib in patients with advanced non-small-cell lung cancer unsuitable for chemotherapy (TOPICAL): a double-blind, placebo-controlled, phase 3 trial. Lancet Oncol. 2012 Nov;13(11):1161-70. doi: 10.1016/S1470-2045(12)70412-6. |
| Lancet32 | Malmstrom A, Henning Gronberg B, Marosi C, Stupp R, Frappaz D, et al. Temozolomide versus standard 6-week radiotherapy versus hypofractionated radiotherapy in patients older than 60 years with glioblastoma: the Nordic randomised, phase 3 trial. Lancet Oncol. 2012 Sep;13(9):916-26. Epub 2012 Aug 8. |
| Lancet33 | Mattano LA Jr, Devidas M, Nachman† JB, Sather HN, Hunger SP, et al. Effect of alternate-week versus continuous dexamethasone scheduling on the risk of osteonecrosis in paediatric patients with acute lymphoblastic leukaemia: results from the CCG-1961 randomised cohort trial. Lancet Oncol. 2012 Sep;13(9):906-15. Epub 2012 Aug 15. |
| Lancet34 | Mees SMD, Algra A, Vanderton WP, van Kooten F, Kuijsten HAJM, et al. Magnesium for aneurysmal subarachnoid haemorrhage (MASH-2): a randomised placebo-controlled trial. Lancet 2012; 380: 44–49. |
| Lancet35 | Moreau C, Delval A, Defebvre L, Dujardin K, Duhamel A, et al. Methylphenidate for gait hypokinesia and freezing in patients with Parkinson's disease undergoing subthalamic stimulation: a multicentre, parallel, randomised, placebo-controlled trial. Lancet Neurol. 2012 Jul;11(7):589-96. doi: 10.1016/S1474-4422(12)70106-0. Epub 2012 Jun 1. |
| Lancet36 | Nogueira RG, Lutsep HL, Gupta R, Jovin TG, Albers GW, et al. Trevo versus Merci retrievers for thrombectomy revascularisation of large vessel occlusions in acute ischaemic stroke (TREVO 2): a randomised trial. Lancet. 2012 Oct 6;380(9849):1231-40. doi: 10.1016/S0140-6736(12)61299-9. Epub 2012 Aug 26. |
| Lancet37 | Papp K, Cather JC, Rosoph L, Sofen H, Langley RG, et al. Efficacy of apremilast in the treatment of moderate to severe psoriasis: a randomised controlled trial. Lancet. 2012 Aug 25;380(9843):738-46. doi: 10.1016/S0140-6736(12)60642-4. Epub 2012 Jun 29. |
| Lancet38 | Paterson AHG, Anderson SJ, Lembersky BC, Fehrenbacher L, Falkson CI, et al. Oral clodronate for adjuvant treatment of operable breast cancer (National Surgical Adjuvant Breast and Bowel Project protocol B-34): a multicentre, placebo-controlled, randomised trial. Lancet Oncol. 2012 Jul;13(7):734-42. doi: 10.1016/S1470-2045(12)70226-7. Epub 2012 Jun 14. |
| Lancet39 | Pavord ID, Korn S, Howarth P, Bleecker ER, Buhl R, et al.Mepolizumab for severe eosinophilic asthma (DREAM): a multicentre, double-blind, placebo-controlled trial. Lancet. 2012 Aug 18;380(9842):651-9. doi: 10.1016/S0140-6736(12)60988-X. |
| Lancet40 | Pettengell R, Coiffier B, Narayanan G, de Mendoza FH, Digumarti R, et al. Pixantrone dimaleate versus other chemotherapeutic agents as a single-agent salvage treatment in patients with relapsed or refractory aggressive non-Hodgkin lymphoma: a phase 3, multicentre, open-label, randomised trial. Lancet Oncol. 2012 Jul;13(7):696-706. doi: 10.1016/S1470-2045(12)70212-7. Epub 2012 May 30. |
| Lancet41 | Pickard R, Lam T, MacLennan G, Starr K, Kilonzo M, et al. Antimicrobial catheters for reduction of symptomatic urinary tract infection in adults requiring short-term catheterisation in hospital: a multicentre randomised controlled trial. Lancet. 2012 Dec 1;380(9857):1927-35. doi: 10.1016/S0140-6736(12)61380-4. Epub 2012 Nov 5. |
| Lancet42 | Pol S, Ghalib RH, Rustgi VK, Martorell C, Everson GR, et al. Daclatasvir for previously untreated chronic hepatitis C genotype-1 infection: a randomised, parallel-group, double-blind, placebo-controlled, dose-finding, phase 2a trial. Lancet Infect Dis. 2012 Sep;12(9):671-7. doi: 10.1016/S1473-3099(12)70138-X. Epub 2012 Jun 18. |
| Lancet43 | Puthanakit T, Saphonn V, Ananworanich J, Kosalaraksa P, Hansudewechakul R, et al. Early versus deferred antiretroviral therapy for children older than 1 year infected with HIV (PREDICT): a multicentre, randomised, open-label trial. Lancet Infect Dis. 2012 Dec;12(12):933-41. doi: 10.1016/S1473-3099(12)70242-6. Epub 2012 Oct 9. |
| Lancet44 | Richmond PC, Marshall HS, Nissen MD, Jiang Q, Jansen KU, et al. Safety, immunogenicity, and tolerability of meningococcal serogroup B bivalent recombinant lipoprotein 2086 vaccine in healthy adolescents: a randomised, single-blind, placebo-controlled, phase 2 trial. Lancet Infect Dis. 2012 Aug;12(8):597-607. doi:10.1016/S1473-3099(12)70087-7. |
| Lancet45 | Rickard CM, Webster J, Wallis MC, Marsh N, McGrail MR, et al. Routine versus clinically indicated replacement of peripheral intravenous catheters: a randomised controlled equivalence trial. Lancet. 2012 Sep 22;380(9847):1066-74. doi: 10.1016/S0140-6736(12)61082-4. |
| Lancet46 | Ridker PM, Pradhan A, MacFadyen JG, Libby P, Glynn RJ. Cardiovascular benefits and diabetes risks of statin therapy in primary prevention: an analysis from the JUPITER trial. Lancet. 2012 Aug 11;380(9841):565-71. doi: 10.1016/S0140-6736(12)61190-8. |
| Lancet47 | Rödel C, Liersch T, Becker H, Fietkau R, Hohenberger W, et al. Preoperative chemradiotherapy and postoperative chemotherapy with fluorouracil and oxaliplatin versus flurouracil alone in locally advanced rectal cancer: initial results of the German CAO/ARO/AIO-04 randomised phase 3 trial. Lancet Oncol. 2012 Jul;13(7):679-87. doi: 10.1016/S1470-2045(12)70187-0. Epub 2012 May 23. |
| Lancet48 | Ryan NM, Birring SS, Gibson PG. Gabapentin for refractory chronic cough: a randomised, double-blind, placebo-controlled trial. Lancet. 2012 Nov 3;380(9853):1583-9. doi: 10.1016/S0140-6736(12)60776-4. Epub 2012 Aug 28. |
| Lancet49 | Sabate M, Cequier A, Iñiguez A, Serra A, Hernandez-Antolin R, et al. Everolimus-eluting stent versus bare-metal stent in ST-segment elevation myocardial infarction (EXAMINATION): 1 year results of a randomised controlled trial. Lancet. 2012 Oct 27;380(9852):1482-90. doi: 10.1016/S0140-6736(12)61223-9. Epub 2012 Sep 3. |
| Lancet50 | Sabchareon A, Wallace D, Sirivichayakul C, Limkittikul K, Chanthavanich P, et al. Protective efficacy of the recombinant, live-attenuated, CYD tetravalent dengue vaccine in Thai schoolchildren: a randomised, controlled phase 2b trial. Lancet. 2012 Nov;380(9853):1559- 67. doi: 10.1016/S0140-6736(12)61428-7. |
| Lancet51 | Sandberg T, Skoog G, Bornefalk Hermansson A, Kahlmeter G, Kuylenstierna N, et al. Ciprofloxacin for 7 days in women with acute pyelonephritis: a randomised, open-label and double-blind, placebo-controlled, non-inferiority trial. Lancet. 2012 Aug 4;380(9840):484-90. doi: 10.1016/S0140-6736(12)60608-4. Epub 2012 Jun 21. |
| Lancet52 | Saver JL, Jahan R, Levy EI, Jovin TG, Baxter B, et al. Solitaire flow restoration device versus the Merci Retriever in patients with acute ischaemic stroke (SWIFT): a randomised, parallel-group, non-inferiority trial. Lancet. 2012 Oct 6;380(9849):1241-9. doi: 10.1016/S0140-6736(12)61384-1. Epub 2012 Aug 26. |
| Lancet53 | Sax PE, Dejesus E, Mills A, Zolopa A, Cohen C, et al. Co-formulated elvitegravir, cobicistat, emtricitabine, and tenofovir versus co-formulated efavirenz, emtricitabine, and tenofavir for initial treatment of HIV-1 infection: a randomised, double-blind, phase 3 trial, analysis of results after 48 weeks. Lancet. 2012 Aug 25;380(9843):730. |
| Lancet54 | Schmitz N, Nickelsen M, Ziepert M, Haenel M, Borchmann P, et al. Conventional chemotherapy (CHOEP-14) with rituximab or high-dose chemotherapy (MegaCHOEP) with rituximab for young, high-risk patients with aggressive B-cell lymphoma: an open-label, randomised, phase 3 trial (DSHNHL 2002-1). ncet Oncol. 2012 Dec;13(12):1250-9. doi: 10.1016/S1470-2045(12)70481-3. Epub 2012 Nov 16. |
| Lancet55 | Simmons RK, Echouffo-Tcheugui JB, Sharp SJ, Sargeant LA, Williams KM, et al. Screening for type 2 diabetes and population mortality over 10 years (ADDITION-Cambridge): a cluster-randomised controlled trial. Lancet. 2012 Nov 17;380(9855):1741-8. doi: 10.1016/S0140-6736(12)61422-6. Epub 2012 Oct 4. |
| Lancet56 | Solomon SD, Zile M, Pieske B, Voors A, Shah A, et al. The angiotensin receptor neprilysin inhibitor LCZ696 in heart failure with preserved ejection fraction: a phase 2 double-blind randomised controlled trial. Lancet. 2012 Oct 20;380(9851):1387-95. doi: 10.1016/S0140-6736(12)61227-6. Epub 2012 Aug 26. |
| Lancet57 | Stein EA, Gipe D, Bergeron J, Gaudet D, Weiss R, Dufour R, Wu R, Pordy R. Effect of a monoclonal antibody to PCSK9, REGN727/SAR236553, to reduce low-density lipoprotein cholesterol in patients with heterozygous familial hypercholesterolaemia on stable statin dose with or without ezetimibe therapy: a phase 2 randomised controlled trial. Lancet. 2012 Jul 7;380(9836):29-36. doi: 10.1016/S0140-6736(12)60771-5. Epub 2012 May 26. |
| Lancet58 | Vellas B, Coley N, Ousset P-J, Berrut G, Dartigues J-F, Dubois B, et al. Long-term use of standardised ginkgo biloba extract for the prevention of Alzheimer's disease (GuidAge): a randomised placebo-controlled trial. Lancet Neurol. 2012 Oct;11(10):851-9. doi: 10.1016/S1474-4422(12)70206-5. Epub 2012 Sep 6. |
| Lancet59 | Walson J, Singa B, Sangaré L, Naulikha J, Piper B, et al. Empiric deworming to delay HIV disease progression in adults with HIV who are ineligible for initiation of antiretroviral treatment (the HEAT study): a multi-site, randomised trial. Lancet Infect Dis. 2012 Dec;12(12):925-32. doi: 10.1016/S1473-3099(12)70207-4. Epub 2012 Sep 10. |
| Lancet60 | Wandt H, Schaefer-Eckart K, Wendelin K, Pilz B, Wilhelm M, et al. Therapeutic platelet transfusion versus routine prophylactic transfusion in patients with haematological malignancies: an open-label, multicentre, randomised study. Lancet. 2012 Oct 13;380(9850):1309-16. doi: 10.1016/S0140-6736(12)60689-8. Epub 2012 Aug 8. |
| Lancet61 | Wick W, Platten M, Meisner C, Felsberg J, Tabatabai G, et al. Temozolomide chemotherapy alone versus radiotherapy alone for malignant astrocytoma in the elderly: the NOA-08 randomised, phase 3 trial. Lancet Oncol. 2012 Jul;13(7):707-15. doi: 10.1016/S1470-2045(12)70164-X. Epub 2012 May 10. |
| Lancet62 | Winblad B, Andreasen N, Minthon L, Floesser A, Imbert G, et al. Safety, tolerability, and antibody response of active AB immunotherapy with CAD106 in patients with Alzheimer's disease: randomised, double-blind, placebo-controlled, first-in-human study. Lancet Neurol. 2012 Jul;11(7):597-604. doi: 10.1016/S1474-4422(12)70140-0. Epub 2012 Jun 6. |
| Lancet63 | Woll PJ, Reichardt P, Le Cesne A, Bonvalot S, Azzarelli A, et al. Adjuvant chemotherapy with doxorubicin, ifosfamide, and lenograstim for resected soft-tissue sarcoma (EORTC 62931): a multicentre randomised controlled trial. Lancet Oncol. 2012 Oct;13(10):1045-54. doi: 10.1016/S1470-2045(12)70346-7. Epub 2012 Sep 4. |
| Lancet64 | Wong C, Jayaram L, Karalus N, Eaton T, Tong C, et al. Azithromycin for prevention of exacerbations in non-cystic fibrosis bronchiectasis (EMBRACE): a randomised, double-blind, placebo-controlled trial. Lancet. 2012 Aug 18;380(9842):660-7. doi: 10.1016/S0140-6736(12)60953-2. |
| Lancet65 | Yoshino T, Mizunuma N, Yamazaki K, Nishina T, Komatsu Y, et al. TAS-102 monotherapy for pretreated metastatic colorectal cancer: a double-blind, randomised, placebo-controlled phase 2 trial. Lancet Oncol. 2012 Oct;13(10):993-1001. doi: 10.1016/S1470-2045(12)70345-5. Epub 2012 Aug 28. |
| Lancet66 | Zinkstok SM, Roos YB, on behalf of the ARTIS investigators. Early administration of aspirin in patients treated with alteplase for acute ischaemic stroke: a randomised controlled trial. Lancet. 2012 Aug 25;380(9843):731-7. doi: 10.1016/S0140-6736(12)60949-0. Epub 2012 Jun 28. |
| NEJM1 | Achan J, Kakuru A, Ikilezi G, Ruel T, Clark TD, Nsanzabana C, et al. Antiretroviral Agents and Prevention of Malaria in HIV-Infected Ugandan Children. N Engl J Med. 2012 Nov 29;367(22):2110-8. doi: 10.1056/NEJMoa1200501. |
| NEJM2 | Afdhal NH, Giannini EG, Tayyab G, Mohsin A, Lee J-W, et al. Eltrombopag before Procedures in Patients with Cirrhosis and Thrombocytopenia. N Engl J Med. 2012 Aug 23;367(8):716-24. doi: 10.1056/NEJMoa1110709. |
| NEJM3 | Agus MSD, Steil GM, Wypij D, Costello JM, Laussen PC, et al. Tight Glycemic Control versus Standard Care after Pediatric Cardiac Surgery. N Engl J Med. 2012 Sep 27;367(13):1208-19. Epub 2012 Sep 7. |
| NEJM4 | Anasetti C, Logan BR, Lee SJ, Waller EK, Weisdorf DJ, et al. Peripheral-Blood Stem Cells versus Bone Marrow from Unrelated Donors. N Engl J Med. 2012 Oct 18;367(16):1487-96. doi: 10.1056/NEJMoa1203517. |
| NEJM5 | Baeten JM, Donnell D, Ndase P, Mugo NR, Campbell JD, et al. Antiretroviral Prophylaxis for HIV Prevention in Heterosexual Men and Women. N Engl J Med. 2012 Aug 2;367(5):399-410. doi: 10.1056/NEJMoa1108524. Epub 2012 Jul 11. |
| NEJM6 | Bart BA, Goldsmith SR, Lee KL, Givertz MM, O'Connor CM, et al. Ultrafiltration in Decompensated Heart Failure with Cardiorenal Syndrome. N Engl J Med. 2012 Dec 13;367(24):2296-304. doi: 10.1056/NEJMoa1210357. Epub 2012 Nov 6. |
| NEJM7 | Boonen S, Reginster J-Y, Kaufman J-M, Lippuner K, Zanchetta J, et al. Fracture Risk and Zoledronic Acid Therapy in Men with Osteoporosis. N Engl J Med. 2012 Nov;367(18):1714-23. doi: 10.1056/NEJMoa1204061. |
| NEJM8 | Brighton TA, Eikelboom JW, Mann K, Mister R, Gallus A, et al. Low-Dose Aspirin for Preventing Recurrent Venous Thromboembolism. N Engl J Med. 2012 Nov 22;367(21):1979-87. doi: 10.1056/NEJMoa1210384. Epub 2012 Nov 4. |
| NEJM9 | Burks AW, Jones SM, Wood RA, Fleischer DM, Sicherer SH, et al. Oral Immunotherapy for Treatment of Egg Allergy in Children. N Engl J Med. 2012 Jul 19;367(3):233-43. doi: 10.1056/NEJMoa1200435. |
| NEJM10 | Chesnut RM, Temkin N, Carney N, Dikmen S, Rondina C, et al. A Trial of Intracranial-Pressure Monitoring in Traumatic Brain Injury. N Engl J Med. 2012 Dec 27;367(26):2471-81. doi: 10.1056/NEJMoa1207363. Epub 2012 Dec 12. |
| NEJM11 | Collet JP, Cuisset T, Rangé G, Cayla G, Elhadad S, et al. Bedside Monitoring to Adjust Antiplatelet Therapy for Coronary Stenting. N Engl J Med. 2012 Nov 29;367(22):2100-9. doi: 10.1056/NEJMoa1209979. Epub 2012 Nov 4. |
| NEJM12 | Cosedis Nielsen J, Johannessen A, Raatikainen P, Hindricks G, Walfridsson H, et al. Radiofrequency Ablation as Initial Therapy in Paroxysmal Atrial Fibrillation. N Engl J Med. 2012 Oct 25;367(17):1587-95. doi: 10.1056/NEJMoa1113566. |
| NEJM13 | Crook JM, O’Callaghan CJ, Duncan G, Dearnaley DP, Higano CS, et al. Intermittent Androgen Suppression for Rising PSA Level after Radiotherapy. N Engl J Med. 2012 Sep 6;367(10):895-903. doi: 10.1056/NEJMoa1201546. |
| NEJM14 | Damme LV, Corneli A, Ahmed K, Agot K, Lombaard J, et al. Preexposure Prophylaxis for HIV Infection among African Women. N Engl J Med. 2012 Aug 2;367(5):411-22. doi: 10.1056/NEJMoa1202614. Epub 2012 Jul 11. |
| NEJM15 | De Bruyne B, Pijls NHJ, Kalesan B, Barbato E, Tonino PAL, et al. Fractional Flow Reserve–Guided PCI versus Medical Therapy in Stable Coronary Disease. N Engl J Med. 2012 Sep 13;367(11):991-1001. doi: 10.1056/NEJMoa1205361. Epub 2012 Aug 27. |
| NEJM16 | de Ruyter JC, Olthof MR, Seidell JC, Katan MB. A Trial of Sugar-free or Sugar-Sweetened Beverages and Body Weight in Children. N Engl J Med. 2012 Oct 11;367(15):1397-406. doi: 10.1056/NEJMoa1203034. Epub 2012 Sep 21. |
| NEJM17 | Devanand DP, Mintzer J, Schultz SK, Andrews HF, Sultzer DL, et al. Relapse Risk after Discontinuation of Risperidone in Alzheimer’s Disease. N Engl J Med. 2012 Oct 18;367(16):1497-507. doi: 10.1056/NEJMoa1114058. |
| NEJM18 | Ebbeling CB, Feldman HA, Chomitz VR, Antonelli TA, Gortmaker SL, et al. A Randomized Trial of Sugar-Sweetened Beverages and Adolescent Body Weight. N Engl J Med. 2012 Oct 11;367(15):1407-16. doi: 10.1056/NEJMoa1203388. Epub 2012 Sep 21. |
| NEJM19 | Euvrard S, Morelon E, Rostaing L, Goffin E, Brocard A, et al. Sirolimus and Secondary Skin-Cancer Prevention in Kidney Transplantation. N Engl J Med. 2012 Jul 26;367(4):329-39. doi: 10.1056/NEJMoa1204166. |
| NEJM20 | EVOLVE Trial Investigators. Effect of Cinacalcet on Cardiovascular Disease in Patients Undergoing Dialysis. N Engl J Med. 2012 Dec 27;367(26):2482-94. doi: 10.1056/NEJMoa1205624. Epub 2012 Nov 3. |
| NEJM21 | Farkouh ME, Domanski M, Sleeper LA, Siami FS, Dangas G, et al. Strategies for Multivessel Revascularization in Patients with Diabetes. N Engl J Med. 2012 Dec 20;367(25):2375-84. doi: 10.1056/NEJMoa1211585. Epub 2012 Nov 4. |
| NEJM22 | Flaherty KT, Infante JR, Daud A, Gonzalez R, Kefford RF, et al. Combined BRAF and MEK Inhibition in Melanoma with BRAF V600 Mutations. N Engl J Med. 2012 Nov;367(18):1714-23. doi: 10.1056/NEJMoa1204061. |
| NEJM23 | Flaherty KT, Robert C, Hersey P, Nathan P, Garbe C, et al. Improved Survival with MEK Inhibition in BRAF-Mutated Melanoma. N Engl J Med. 2012 Jul 12;367(2):107-14. doi: 10.1056/NEJMoa1203421. Epub 2012 Jun 4. |
| NEJM24 | Fleischmann R, Kremer J, Cush J, Schulze-Koops H, Connell CA, et al. Placebo-Controlled Trial of Tofacitinib Monotherapy in Rheumatoid Arthritis. N Engl J Med. 2012 Aug 9;367(6):495-507. doi: 10.1056/NEJMoa1109071. |
| NEJM25 | Fox RJ, Miller DH, Phillips JT, Hutchinson M, Havrdova E, et al. Placebo-Controlled Phase 3 Study of Oral BG-12 or Glatiramer in Multiple Sclerosis. N Engl J Med. 2012 Sep 20;367(12):1087-97. |
| NEJM26 | Gold R, Kappos L, Arnold DL, Bar-Or A, Giovannoni G, et al. Placebo-Controlled Phase 3 Study of Oral BG-12 for Relapsing Multiple Sclerosis. N Engl J Med. 2012 Sep 20;367(12):1098-107. |
| NEJM27 | Hoffmann U, Truong QA, Schoenfeld DA, Chou ET, Woodard PK, et al.Coronary CT Angiography versus Standard Evaluation in Acute Chest Pain. N Engl J Med. 2012 Jul 26;367(4):299-308. doi: 10.1056/NEJMoa1201161. |
| NEJM28 | Kelly HW, Sternberg AL, Lescher R, Fuhlbrigge AL, Williams P, et al. Effect of inhaled glucocorticoids in childhood on adult height. N Engl J Med. 2012 Sep 6;367(10):904-12. doi: 10.1056/NEJMoa1203229. Epub 2012 Sep 3. |
| NEJM29 | Kerstjens HAM, Engel M, Dahl R, Paggiaro P, Beck E, et al. Tiotropium in Asthma Poorly Controlled with Standard Combination Therapy. N Engl J Med. 2012 Sep 27;367(13):1198-207. Epub 2012 Sep 2. |
| NEJM30 | Kluin-Nelemans HC, Hoster E, Hermine O, Walewski J, Trneny M, et al. Treatment of Older Patients with Mantle-Cell Lymphoma. N Engl J Med. 2012 Aug 9;367(6):520-31. doi: 10.1056/NEJMoa1200920. |
| NEJM31 | Lederle FA, Freischlag JA, Kyriakides TC, Matsumura JS, Padberg FT, et al. Long-term comparison of endovascular and open repair of abdominal aortic aneurysm. N Engl J Med. 2012 Nov 22;367(21):1988-97. doi: 10.1056/NEJMoa1207481. |
| NEJM32 | Lee M, Lee J, Carroll MW, Choi H, Min S,et al. Linezolid for Treatment of Chronic Extensively Drug-Resistant Tuberculosis. N Engl J Med. 2012 Oct 18;367(16):1508-18. doi: 10.1056/NEJMoa1201964. |
| NEJM33 | Mehta RS, Barlow WE, Albain KS, Vandenberg TA, Dakhil SR, et al. Combination Anastrozole and Fulvestrant in Metastatic Breast Cancer. N Engl J Med. 2012 Aug 2;367(5):435-44. doi: 10.1056/NEJMoa1201622. |
| NEJM34 | Moss AJ, Schuger C, Beck CA, Brown MW, Cannom DS, et al. Reduction in Inappropriate Therapy and Mortality through ICD Programming. N Engl J Med. 2012 Dec 13;367(24):2275-83. doi: 10.1056/NEJMoa1211107. Epub 2012 Nov 6. |
| NEJM35 | Myburgh JA, Finfer S, Bellomo R, Billot L, Cass A, et al. Hydroxyethyl Starch or Saline for Fluid Resuscitation in Intensive Care. N Engl J Med. 2012 Nov 15;367(20):1901-11. doi: 10.1056/NEJMoa1209759. Epub 2012 Oct 17. |
| NEJM36 | ORIGIN Trial Investigators. n–3 Fatty Acids and Cardiovascular Outcomes in Patients with Dysglycemia. N Engl J Med. 2012 Jul 26;367(4):309-18. doi: 10.1056/NEJMoa1203859. Epub 2012 Jun 11. |
| NEJM37 | Pariser DM, Meinking TL, Bell M, Ryan WG. Topical 0.5% Ivermectin Lotion for Treatment of Head Lice. N Engl J Med. 2012 Nov;367(18):1687-93. doi: 10.1056/NEJMoa1200107. |
| NEJM38 | Parving H-H, Brenner B M, McMurray JJV, de Zeeuw D, Haffner SM, et al. Cardiorenal End Points in a Trial of Aliskiren for Type 2 Diabetes. N Engl J Med. 2012 Dec 6;367(23):2204-13. doi: 10.1056/NEJMoa1208799. Epub 2012 Nov 3. |
| NEJM39 | Perner A, Haase N, Guttormsen AB, Tenhunen J, Klemenzson G, et al. Hydroxyethyl Starch 130/0.42 versus Ringer’s Acetate in Severe Sepsis. N Engl J Med. 2012 Jul 12;367(2):124-34. doi: 10.1056/NEJMoa1204242. Epub 2012 Jun 27. |
| NEJM40 | Roe MT, Armstrong PW, Fox KAA, White HD, Prabhakaran D, et al. Prasugrel versus Clopidogrel for Acute Coronary Syndromes without Revascularization. N Engl J Med. 2012 Oct 4;367(14):1297-309. doi: 10.1056/NEJMoa1205512. Epub 2012 Aug 25. |
| NEJM41 | Roth EM, McKenney JM, Hanotin C, Asset G, Stein EA. Atorvastatin with or without an Antibody to PCSK9 in Primary Hypercholesterolemia. N Engl J Med. 2012 Nov 15;367(20):1891-900. doi: 10.1056/NEJMoa1201832. Epub 2012 Oct 31. |
| NEJM42 | Ruperto N, Brunner HI, Quartier P, Constantin T, Wulffraat N, et al. Two Randomized Trials of Canakinumab in Systemic Juvenile Idiopathic Arthritis. N Engl J Med. 2012 Dec 20;367(25):2396-406. doi: 10.1056/NEJMoa1205099. |
| NEJM43 | Sandborn WJ, Gasink C, Gao L-L, Blank MA, Johanns J, et al. Ustekinumab Induction and Maintenance Therapy in Refractory Crohn's Disease. N Engl J Med. 2012 Oct 18;367(16):1519-28. doi: 10.1056/NEJMoa1203572. |
| NEJM44 | Sandborn WJ, Ghosh S, Panes J, Vranic I, Su C, et al. Tofacitinib, an Oral Janus Kinase Inhibitor, in Active Ulcerative Colitis. N Engl J Med. 2012 Aug 16;367(7):616-24. doi: 10.1056/NEJMoa1112168. |
| NEJM45 | Scher HI, Fizazi K, Saad F, Taplin M-E, Sternberg CN, et al. Increased Survival with Enzalutamide in Prostate Cancer after Chemotherapy. N Engl J Med. 2012 Sep 27;367(13):1187-97. Epub 2012 Aug 15. |
| NEJM46 | Stalmans P, Benz MS, Gandorfer A, Kampik A, Girach A, et al. Enzymatic Vitreolysis with Ocriplasmin for Vitreomacular Traction and Macular Holes. N Engl J Med. 2012 Aug 16;367(7):606-15. doi: 10.1056/NEJMoa1110823. |
| NEJM47 | The SPS3 Investigators. Effects of Clopidogrel Added to Aspirin in Patients with Recent Lacunar Stroke. N Engl J Med. 2012 Aug 30;367(9):817-25. doi: 10.1056/NEJMoa1204133. |
| NEJM48 | Thiele H, Zeymer U, Neumann F-J, Ferenc M, Olbrich H-G, et al. Intraaortic Balloon Support for Myocardial Infarction with Cardiogenic Shock. N Engl J Med. 2012 Oct 4;367(14):1287-96. doi: 10.1056/NEJMoa1208410. Epub 2012 Aug 26. |
| NEJM49 | Thigpen MC, Kebaabetswe PM, Paxton LA, Smith DK, Rose CE, et al. Antiretroviral Preexposure Prophylaxis for Heterosexual HIV Transmission in Botswana. N Engl J Med. 2012 Aug 2;367(5):423-34. doi: 10.1056/NEJMoa1110711. Epub 2012 Jul 11. |
| NEJM50 | Torres VE, Chapman AB, Devuyst O, Gansevoort RT, Grantham JI, et al. Tolvaptan in Patients with Autosomal Dominant Polycystic Kidney Disease. N Engl J Med. 2012 Dec 20;367(25):2407-18. doi: 10.1056/NEJMoa1205511. Epub 2012 Nov 3. |
| NEJM51 | van Vollenhoven RF, Fleischmann R, Cohen S, Lee EB, García Meijide JA, et al. Tofacitinib or Adalimumab versus Placebo in Rheumatoid Arthritis. N Engl J Med. 2012 Aug 9;367(6):508-19. doi: 10.1056/NEJMoa1112072. |
| NEJM52 | Vaucher YE, Peralta-Carcelen M, Finer NN, Carlo WA, Gantz MG, et al. Neurodevelopmental Outcomes in the Early CPAP and Pulse Oximetry Trial. N Engl J Med. 2012 Dec 27;367(26):2495-504. doi: 10.1056/NEJMoa1208506. |
| NEJM53 | Verma S, Miles D, Gianni L, Krop IE, Welslau M, et al. Trastuzumab Emtansine for HER2-Positive Advanced Breast Cancer. N Engl J Med. 2012 Nov 8;367(19):1783-91. doi: 10.1056/NEJMoa1209124. Epub 2012 Oct 1. |
| NEJM54 | Visco AG, Brubaker L, Richter HE, Nygaard I, Paraiso MFR, et al. Anticholinergic Therapy vs. OnabotulinumtoxinA for Urgency Urinary Incontinence. N Engl J Med. 2012 Nov 8;367(19):1803-13. doi: 10.1056/NEJMoa1208872. Epub 2012 Oct 4. |
| NEJM55 | Wilt TJ, Brawer MK, Jones KM, Barry MJ, Aronson WJ, et al. Radical Prostatectomy versus Observation for Localized Prostate Cancer. N Engl J Med. 2012 Jul 19;367(3):203-13. doi: 10.1056/NEJMoa1113162. |
